# ﻿The first mitogenomes of the subfamily Epipleminae (Lepidoptera, Uraniidae) and phylogenetic analysis of Macroheterocera

**DOI:** 10.3897/zookeys.1255.164711

**Published:** 2025-10-15

**Authors:** Yanpeng Cai, Aihui Yin

**Affiliations:** 1 Molecular Diagnostic Research Center, Guizhou University of Traditional Chinese Medicine, Guiyang 550025, Guizhou, China Guizhou University of Traditional Chinese Medicine Guiyang China

**Keywords:** 16S rRNA, Epipleminae, Macroheterocera, mitogenome, phylogeny, Uraniidae

## Abstract

The subfamily Epipleminae, the largest group within Uraniidae (Lepidoptera, Macroheterocera, Geometroidea), comprises small, nocturnal moths primarily distributed in tropical regions. Taxonomic and molecular phylogenetic studies of Epipleminae have long been challenging, and this group remains genetically understudied, with no mitochondrial genomes (mitogenomes) reported to date, despite their wide utility in phylogenetic research. Here, we sequenced, assembled, and annotated the first complete mitogenomes of four epiplemine species: *Dysaethria flavistriga* (15,404 bp), *Monobolodes prunaria* (15,258 bp), *Phazaca alikangensis* (15,482 bp), and *Warreniplema fumicosta* (15,467 bp), using high-throughput sequencing technology. These mitogenomes exhibit typical gene arrangement of ditrysian Lepidoptera, along with distinctive features such as rare (TA)n microsatellite repeats in the 16S rRNA. Most protein-coding genes (PCGs) initiate with standard ATN start codons and terminate with TAA or a single T residue. Codon usage analysis revealed UUA (Leu2), AUU (Ile), UUU (Phe), AUA (Met), and AAU (Asn) as the five most frequently used codons. All tRNAs display canonical cloverleaf secondary structures, except for trnS1, which lacks the DHU arm. Comprehensive phylogenetic analyses that incorporated existing macroheteroceran mitogenomic data provided robust support for the placement of Epipleminae under Uraniidae, offered the first mitogenome-based evidence supporting the monophyly of Geometroidea on family level, and strongly supported a sister relationship between Geometroidea and Noctuoidea.

## ﻿Introduction

The insect mitochondrial genome (mitogenome) is a double-stranded closed loop DNA molecule, measuring 15–18 kb in size. It encodes a pack of 37 genes, including 13 protein-coding genes, 22 transfer RNA genes, and two ribosomal RNA genes, which exhibit strong conservation across bilaterian metazoans (Boore 1999; Cameron 2014). In addition to the genes, the mitogenome typically contains at least one AT-rich noncoding control region of variable lengths, which serves critical regulatory functions in transcription and replication processes (Clayton 1992; Cameron 2014). The mitogenome’s compact size and high copy number render it significantly easier for lab extraction compared with the nuclear genome (Cameron 2014; Zhang et al. 2023). In recent years, driven by advancements in next-generation sequencing (NGS) technology, insect mitogenome sequence data have indeed been rapidly accumulated and are now widely applied in evolutionary studies, particularly phylogenetics (Curole and Kocher 1999; Cameron 2014; Yang et al. 2019a).

The mega-diverse Macroheterocera represents the high-level phylogenetic lineage of the “large moths” in Lepidoptera. It currently includes six superfamilies (Bombycoidea, Drepanoidea, Geometroidea, Lasiocampoidea, Mimallonoidea, and Noctuoidea), and consists of more than 72,400 described species (van Nieukerken et al. 2011; Mitter et al. 2017; Rota et al. 2022). A substantial number of molecular phylogenetic studies involving Macroheterocera have been carried out in the recent two decades (e.g. Mutanen et al. 2010; Bazinet et al. 2013; Heikkilä et al. 2015; Kawahara et al. 2019; Cheng et al. 2022). It is shown that Bombycoidea, Lasiocampoidea, Geometroidea and Noctuoidea are no doubt closely related within Macroheterocera, although their interrelationships are still debatable (Cheng et al. 2022; Rota et al. 2022). Mimallonoidea and Drepanoidea, on the other hand, are highly labile regarding their phylogenetic affinities. In most previous studies, they are recovered and accepted as two basal lineages that successively branched off from the remaining macroheterocerans (Bazinet et al. 2013; Timmermans et al. 2014; Kawahara et al. 2019; Yang et al. 2019b; Mayer et al. 2021; Rota et al. 2022). Yet in some studies, Mimallonoidea has been found to form a subclade within Drepanoidea at the base of Macroheterocera (Regier et al. 2009; Regier et al. 2013), or group together with Bombycoidea + Lasiocampoidea (Wang et al. 2019; Chen et al. 2022), or even be placed far apart from other macroheterocerans (Mutanen et al. 2010), while Drepanoidea’s placement within Macroheterocera is even more unstable, with its phylogenetic position conflicting across analyses (Mutanen et al. 2010; Heikkilä et al. 2015; Wang et al. 2019; Cheng et al. 2022; Zheng et al. 2022).

Uraniidae is a family within the superfamily Geometroidea of Macroheterocera, and comprises ~700 species in ~90 genera (Minet and Scoble 1998; van Nieukerken et al. 2011). This family is distributed worldwide, with its members predominantly found in the pantropical regions (Holloway 1998). Uraniids differ from other Lepidopterans by having unique sexually dimorphic tympanal organs (Scoble 1992). They are delicate, small to large, broad-winged moths; their hindwing termens are often angulate or with tail-shaped projections (Minet and Scoble 1998). Some swallowtail-like diurnal species are renowned for their brilliant iridescent coloration and prominently tailed hindwings (Scoble 1992).

Epipleminae, the most speciose subfamily of the four subfamilies (the other three: Auzeinae, Microniinae, and Uraniinae) in Uraniidae (Minet and Scoble 1998), was established by Hampson (1892) originally as a family based on very simple diagnostic characters of the wing venation. Modern phylogenetic revisions have redefined its status as a subfamily under Uraniidae by the synapomorphic character of the tympanal organ (Sick 1937; Minet 1983, 1986). It currently includes over 600 species in ~70 genera (Sohn and Yen 2005), and is primarily distributed in pantropical montane zones (Holloway 1998; Smetacek 2005). Compared with the other three subfamilies, the epiplemines are generally small, drab-colored nocturnal moths (Scoble 1992; Minet and Scoble 1998). The resting posture is a distinctive feature of this subfamily (Sohn and Yen 2005). Typically, their hindwings are folded along the abdomen, while forewings are extended horizontally, either flat or longitudinally rolled, the entire body forms a T-shaped dorsal profile when viewed from above (Minet and Scoble 1998; Sohn and Yen 2005). Like many early-established lepidopteran taxa, the generic taxonomy of Epipleminae has remained highly unsatisfactory since its establishment, and a robust phylogenetic framework for this subfamily is urgently needed as well (Holloway 1998; Sohn and Yen 2005). Unfortunately, no molecular phylogenetic study focusing on Epipleminae or Uraniidae has been published to date. Despite the significant utility of mitogenomes in phylogenetic research, only three mitochondrial genome sequences have been published for Uraniidae (as of 8 July 2025), covering just two species from two subfamilies, Microniinae and Uraniinae. Notably, no mitogenome sequences are available for Epipleminae, the largest subfamily within Uraniidae.

Accordingly, in this study, we present four complete mitogenome sequences for Epipleminae for the first time. Our focal species include *Dysaethria flavistriga*, *Monobolodes prunaria*, *Phazaca alikangensis*, and *Warreniplema fumicosta*, of which the specimens were all collected from Guizhou, China. We provide a detailed description and comparative analysis of these mitogenomes. Additionally, we explore the phylogenetic positions of Epipleminae and Uraniidae, and further reconstruct the phylogeny of Macroheterocera using available mitogenome data.

## ﻿Materials and methods

### ﻿Sample collection and identification

Adult specimens of the four epiplemine species were captured by light trap from Baiyan Village (25°21'47"N, 107°56'5"E, 748 m elevation), Maolan National Nature Reserve, Libo County, Guizhou, China on 8 July 2024. The specimens were pinned and dried, and their genitalia were dissected and slide-mounted for examination. Identification was based on external morphology and genitalia characters, following taxonomic references (Holloway 1998; Sohn and Yen 2005; Choi et al. 2024).

### ﻿DNA extraction and sequencing

Total DNA was extracted from the thoracic muscles of the dry specimens using the MagicMag Genomic DNA Micro Kit (Sangon Biotech Co., Shanghai, China) following the manufacturer’s protocol. For each sample, 0.2 μg DNA was fragmented by sonication to an average size of 350 bp. The fragmented DNA was then subjected to high-throughput pair-ended sequencing (PE150) on Illumina NovaSeq 6000 platform at Novogene Bioinformatics Technology Co., Ltd (Tianjin, China).

### ﻿Mitogenome assembly, annotation, and analysis

The raw data were processed using Fastp v. 0.19.7 (Chen et al. 2018) to remove low-quality reads, yielding ~3 Gb of clean data for each sample. The complete mitogenomes were assembled de novo using MitoZ v.3.6 (Meng et al. 2019) and SPAdes v. 4.0.0 (Prjibelski et al. 2020). The assembled sequences were polished with Pilon v. 1.24 (Walker et al. 2014), and annotated using MitoZ software and MITOS2 online service (Galaxy v. 2.1.9) (Al Arab et al. 2017; Donath et al. 2019), followed by manual verification. Final mitogenome maps were generated using Organellar Genome DRAW (OGDRAW) (Lohse et al. 2013).

The mitogenomes were then uploaded to GenBank. The standard 658 bp *COX1* barcodes were extracted from the mitogenome sequences and uploaded to the BOLD system. The corresponding GenBank accession numbers and BOLD sample IDs are listed in Table 1.

**Table 1.** The GenBank accession numbers and BOLD sample IDs of the four epiplemine species.

Species	Accession numbers (GenBank)	Sample IDs (BOLD)
*Dysaethria flavistriga*	PV151521	GZUTCM:SWD
*Monobolodes prunaria*	PV151522	GZUTCM:SWM
*Phazaca alikangensis*	PV151523	GZUTCM:SWP
*Warreniplema fumicosta*	PV151524	GZUTCM:SWW

The base composition of the mitogenomes was analyzed using MEGA v. 11.0.13 (Tamura et al. 2021). Nucleotide composition bias between the two strands was calculated using the formulas proposed by Perna and Kocher (1995): AT-skew = (A-T)/(A+T) and GC-skew = (G-C)/(G+C). Relative synonymous codon usage (RSCU) diagrams for the PCGs were generated and analyzed using PhyloSuite v. 1.2.3 (Zhang et al. 2020; Xiang et al. 2023). The non-synonymous substitution rate (Ka), synonymous substitution rate (Ks), and their ratio (Ka/Ks) for each PCG in Epipleminae were calculated through DnaSP v. 5.10.01 (Librado and Rozas 2009).

The secondary structures of the tRNAs were predicted via MITOS2 online service, while rRNA secondary structures were predicted with R2DT v. 1.3 (Sweeney et al. 2021), both with manual verification. Tandem repeat elements in the control regions were identified using Tandem Repeats Finder (https://tandem.bu.edu/trf/home) (Benson 1999).

### ﻿Phylogenetic analyses

To determine the phylogenetic positions of Epipleminae and Uraniidae, and to reconstruct the phylogeny of Macroheterocera, we aimed to maximize taxonomic representation at the family and subfamily level. We downloaded mitogenomic sequences for 34 species from GenBank, representing 18 families within Macroheterocera and four outgroup families (Table 2). In addition, we assembled four novel mitogenomic sequences for Epipleminae in this study. For three other families (Cimeliidae, Pseudobistonidae, and Sematuridae) and one subfamily (Cyclidiinae of Drepanidae), which lack publicly available mitogenomic data but have accessible genomic/transcriptomic SRA files (NCBI), we assembled mitogenomic sequences of five species (Table 2). However, due to the low quality of the SRA data, the three sequences for Cimeliidae, Pseudobistonidae, and Cyclidiinae were only partially assembled.

**Table 2.** Basic information of the mitogenomes used for the phylogenetic analyses in this study. Accession numbers of newly sequenced species are highlighted in bold.

Superfamily	Family	Subfamily	Species	Accession/SRA number
**Ingroup**				
Bombycoidea	Bombycidae	Bombycinae	*Bombyx mandarina*	AB070263
	Brahmaeidae	–	*Brahmaea hearseyi*	KU884326
	Endromidae	–	*Andraca theae*	KX365419
	Eupterotidae	–	*Ganisa cyanogrisea*	MF100140
	Saturniidae	Saturniinae	*Antheraea pernyi*	MK920216
	Sphingidae	Macroglossinae	*Theretra clotho*	MZ562564
Drepanoidea	Cimeliidae	–	*Axia margarita*	SRR1006157
	Doidae	–	*Doa* sp.	KJ508058
	Drepanidae	Cyclidiinae	*Cyclidia substigmaria*	SRR1021608
		Drepaninae	*Pseudalbara parvula*	MZ823341
		Oretinae	*Oreta* sp.	MZ823343
		Thyatirinae	*Tethea albicostata*	OK149234
Geometroidea	Epicopeiidae	–	*Epicopeia hainesii*	MK033610
	Geometridae	Alsophilinae	*Alsophila aescularia*	OX276387
		Ennominae	*Ectropis grisescens*	MN792921
		Geometrinae	*Iotaphora admirabilis*	MK903032
		Larentiinae	*Hydrelia parvulata*	MN962739
		Sterrhinae	*Idaea salutaria*	MK122626
	Pseudobistonidae	Pseudobistoninae	*Pseudobiston pinratanai*	SRR11994912, SRR13187626
	Sematuridae	–	*Homidiana leachi*	SRR27474561
		–	*Mania lunus*	SRR27474554, SRR27474556
	Uraniidae	Epipleminae	*Dysaethria flavistriga*	**PV151521**
			*Monobolodes prunaria*	**PV151522**
			*Phazaca alikangensis*	**PV151523**
			*Warreniplema fumicosta*	**PV151524**
		Microniinae	*Acropteris iphiata*	MN093120
		Uraniinae	*Lyssa zampa*	MZ713634
Lasiocampoidea	Lasiocampidae	Chaetomalachiinae	*Euthrix laeta*	KU870700
		Pinarinae	*Trabala vishnou*	MZ927091
Mimallonoidea	Mimallonidae	–	*Lacosoma valva*	KJ508050
Noctuoidea	Erebidae	Arctiinae	*Brunia dorsalis*	MN635735
		Erebinae	*Erebus caprimulgus*	MZ959074
		Lymantriinae	*Dasychira tristis*	MZ520324
	Euteliidae	Euteliinae	*Eutelia adulatricoides*	KJ185131
	Noctuidae	Amphipyrinae	*Spodoptera frugiperda*	KU877172
		Dilobinae	*Diloba caeruleocephala*	OX381635
		Eustrotiinae	*Maliattha signifera*	OQ111926
	Nolidae	Eariadinae	*Earias clorana*	OK235312
	Notodontidae	Dudusinae	*Dudusa sphingiformis*	MW788876
**Outgroup**				
Calliduloidea	Callidulidae	–	*Pterodecta felderi*	MT370823
Hyblaeoidea	Hyblaeidae	–	*Hyblaea puera*	MW885970
Papilionoidea	Papilionidae	Parnassiinae	*Luehdorfia chinensis*	KM453725
Pyraloidea	Pyralidae	Phycitinae	*Plodia interpunctella*	KT428892

In our phylogenetic analyses, multi-gene and multi-method approaches were employed to mitigate single-locus bias, resolve topological conflicts, and strengthen nodal support, thereby ensuring robust phylogenetic hypotheses. Consequently, a total of ten phylogenetic trees were reconstructed using Maximum Likelihood (ML) and Bayesian Inference (BI) methods based on five data matrices: (1) “PCG123R” matrix: All three codon positions of 13 PCGs plus two rRNA genes (13,214 sites); (2) “PCG123” matrix: All three codon positions of 13 PCGs (11,163 sites); (3) “PCG12R” matrix: The 1^st^ and 2^nd^ codon positions of PCGs plus two rRNA genes (9,493 sites); (4) “PCG12” matrix: The 1^st^ and 2^nd^ codon positions of PCGs (7,442 sites); (5) “AA” matrix: Amino acid sequences translated from 13 PCGs (3,721 sites).

The DNA sequences of each gene were aligned separately using MAFFT v. 7.52 (Katoh and Standley 2013) with the L-INS-i algorithm. Poorly aligned positions were removed by trimAI v. 1.4.1 (Capella-Gutiérrez et al. 2009). Individual genes were then concatenated in MEGA 11 software to generate the five datasets mentioned above. Compositional heterogeneity among sequences was analyzed separately for each dataset with AliGROOVE v. 1.0.8 (Kück et al. 2014).

For the AA matrix, the site-heterogeneous substitution model (CAT + GTR) was used in the BI analysis. This model was chosen because it demonstrated better performance against systematic errors and long-branch attraction (LBA) artifacts compared to site-homogeneous models, despite its higher computational demand. For all other tree-building analyses, site-homogeneous models were applied, and the optimal partitioning schemes and corresponding best-fit substitution models were determined using ModelFinder v. 2.2.2 (Kalyaanamoorthy et al. 2017) implemented in IQ-TREE2 v. 2.3.6 (Minh et al. 2020) (Suppl. material 2: tables S1–S9).

The ML trees were constructed in IQ-TREE2 software with 10,000 ultrafast bootstraps (BS) (Hoang et al. 2018). The BI tree from AA matrix was generated via PhyloBayes-MPI v. 1.8c (Lartillot et al. 2013), with two independent searches of 50,000 generations until convergence (maxdiff < 0.1). The BI trees for the other four datasets were generated via MrBayes v. 3.2.7 (Ronquist et al. 2012), with four independent chains run for 5–7 million generations until the average standard deviation of split frequency dropped below 0.01. In each BI analysis, the initial 25% of trees were discarded as burn-in, and consensus trees with posterior probabilities (PP) for each node were computed from the remaining trees. All phylogenetic trees were visualized in FigTree v. 1.4.4 (Rambaut et al. 2018).

## ﻿Results and discussion

### ﻿Mitogenome structure and organization

**Figure 1.**
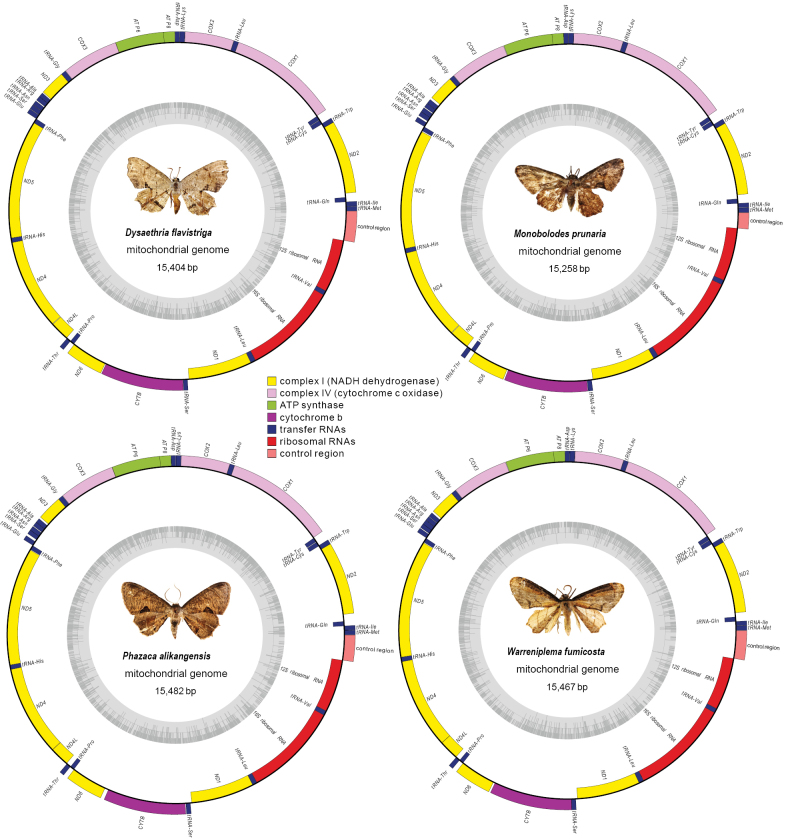
Circular structures of the mitochondrial genomes of four newly sequenced epiplemine species. Genes are represented with different color blocks. Colored blocks outside of each ring indicate that the genes are on the majority strand, while colored blocks within the rings indicate that the genes are located on the minority strand.

The complete mitogenomes of *D. flavistriga* (length: 15,404 bp), *M. prunaria* (length: 15,258 bp), *P. alikangensis* (length: 15,482 bp), and *W. fumicosta* (length: 15,467 bp) were sequenced and annotated. As expected, these epiplemine species shared the typical composition of 37 genes (13 PCGs, 22 tRNA genes, and two rRNA genes) and one A+T-rich control region with other metazoan animals (Wolstenholme 1992) (Fig. 1). Their gene arrangement pattern followed that of most other ditrysian moths (Cao et al. 2012; Park et al. 2016), with nine PCGs and 14 tRNAs encoded on the majority (J) strand, while the remaining four PCGs, eight tRNAs and the two rRNAs on the minority (N) strand (Suppl. material 2: table S10).

The mitogenomes of the four epiplemine species exhibited a distinct overall A + T nucleotide bias (80.8% to 81.2%). This high A+T content varied slightly among different types of genes, being most pronounced in the control region (93.7% to 95.6%), followed by rRNAs (84.9% to 85.7%), tRNAs (81.5% to 82.1%), and lowest in PCGs (79.0% to 79.8%) (Table 3). The AT-skews of the mitogenomes were not significant either across the entire sequences or in different coding regions (-0.085 to 0.044) (Table 3). In contrast, the GC-skew values were much greater and consistently negative, both across the whole mitogenomes and in different coding regions (-0.667 to -0.091) (Table 3).

**Table 3.** Base composition in different regions in the mitochondrial genomes of four epiplemine species: *D. flavistriga*/*M. prunaria*/*P. alikangensis*/*W. fumicosta*.

Feature	Size (bp)	A+T%	AT-skew	GC-skew
Whole mitogenome	15404/15258/ 15482/15467	81.2%/81.2%/ 80.8%/81.2%	0.007/-0.0004/ 0.020/0.021	-0.187/-0.172/ -0.200/-0.195
PCGs	11200/11200/ 11202/11199	79.7%/79.8%/ 79.0%/79.4%	0.009/0.003/ 0.018/0.019	-0.170/-0.152/ -0.181/-0.176
rRNA genes	2177/2179/ 2195/2237	85.2%/85.4%/ 84.9%/85.7%	0.009/-0.009/ 0.015/0.027	-0.344/-0.342/ -0.372/-0.352
tRNA genes	1467/1459/ 1461/1458	82.0%/82.0%/ 82.1%/81.5%	0.024/0.038/ 0.044/0.034	-0.091/-0.125/ -0.115/-0.122
Control region	431/226/371/411	93.7%/93.8%/ 95.4%/95.6%	-0.059/-0.085/ -0.028/0.038	-0.630/-0.143/ -0.412/-0.667

Gene overlaps and intergenic spacers were identified in the mitogenomes of the four epiplemine species: *D. flavistriga* (6 overlaps, 15 spacers), *M. prunaria* (5 overlaps, 18 spacers), *P. alikangensis* (8 overlaps, 15 spacers), and *W. fumicosta* (8 overlaps, 14 spacers) (Suppl. material 2: table S10). The gene overlaps ranged in size from 1 to 8 bp, with the longest overlap (8 bp, sequence: AAGCCTTA) occurring between the *trnW* and *trnC* genes in all epiplemine species (Suppl. material 2: table S10). The intergenic spacers ranged in size from 1 to 100 bp, with the longest spacer located between the *trnQ* and *ND2* genes, measuring 56 bp in *D. flavistriga*, 54 bp in *M. prunaria*, 100 bp in *P. alikangensis*, and 57 bp in *W. fumicosta* (Suppl. material 2: table S10). A long noncoding spacer between the *trnQ* and *ND2* genes appears to be a common feature among all lepidopterans.

### ﻿PCGs and codon usage

The total lengths of the 13 PCGs in the mitogenomes of *D. flavistriga*, *M. prunaria*, *P. alikangensis* and *W. fumicosta* were 11,200 bp, 11,200 bp, 11,202 and 11,199 bp respectively. Most PCGs in the four mitogenomes used the typical ATN start codon for initiation (Suppl. material 2: table S10). However, several genes exhibited unconventional start codons: The putative codon CGA was used in *COX1* in all four species; GTG was used in *ATP6* in *D. flavistriga*; and TTG was used in *ND1* in *M. prunaria*, *P. alikangensis* and *W. fumicosta* (Suppl. material 2: table S10). Regarding stop codons, most PCGs in the four mitogenomes terminated with either the standard stop codon TAA (observed in *ND2*, *ATP8*, *ATP6*, *COX3*, *ND3*, *ND4L*, *ND6*, and *CYTB* in all four species; *ND5* in three species; *ND4* and *ND1* in one species), or a single T residue (observed in *COX1* and *COX2* in all four species; *ND4* in three species; *ND1* in two species; *ND5* in one species) (Suppl. material 2: table S10). The only exception was *ND1* gene in *W. fumicosta*, which terminated with TAG (Suppl. material 2: table S10).

**Figure 2.**
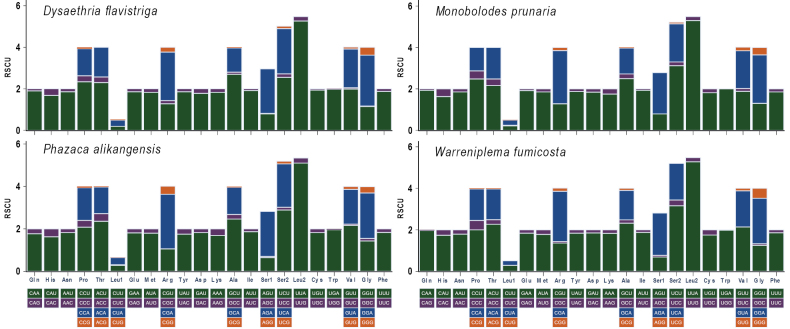
Relative synonymous codon usages (RSCU) of four newly sequenced epiplemine species. Codon families are indicated below the X-axes.

The relative synonymous codon usages (RSCU) for the four mitogenomes are shown in Fig. 2. The analysis revealed that the five most frequently used codons were UUA (Leu2), AUU (Ile), UUU (Phe), AUA (Met), and AAU (Asn). In contrast, several codons were absent in these mitogenomes: ACG (Thr), AGG (Ser1), and CUC (Leu1) in *D. flavistriga*; ACG (Thr), AGC (Ser1), AGG (Ser1), CCG (Pro), CGC (Arg), GGC (Gly), and UGG (Trp) in *M. prunaria*; AGG (Ser1) and CGC (Arg) in *P. alikangensis*; AGG (Ser1), CUC (Leu1), CUG (Leu1), GUC (Val) and UCG (Ser2) in *W. fumicosta*. AGG (Ser1) was the only codon absent in all four species. In conclusion, synonymous codons ending with A or T were strongly preferred over those ending with C or G when encoding the same amino acid.

**Figure 3.**
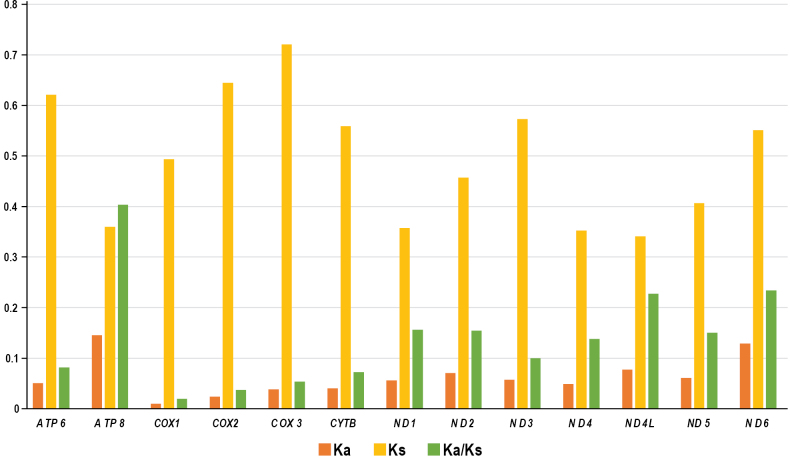
The Ka, Ks, and Ka/Ks values of the 13 mitochondrial PCGs in Epipleminae.

The synonymous substitution rates (Ks), non-synonymous substitution rates (Ka), and the Ka/Ks ratios of the 13 PCGs in Epipleminae were calculated (Fig. 3). The Ka/Ks values were used to estimate the natural selective pressures acting on the PCGs within the population (Hurst 2002). In this study, the Ka/Ks values for all 13 PCGs were less than one (0.02 in *COX1* to 0.40 in *ATP8*), indicating varying levels of purifying selection across these genes (weakest in *COX1* and strongest in *ATP8*).

### ﻿tRNA genes, rRNA genes, and the control region

The total lengths of the 22 tRNA genes in the mitogenomes of *D. flavistriga*, *M. prunaria*, *P. alikangensis* and *W. fumicosta* were 1,467 bp, 1,459 bp, 1,461 and 1,458 bp, respectively. The lengths of individual tRNA genes ranged from 62 bp (the shortest, *trnS1*, in *P. alikangensis* and *W. fumicosta*) to73 bp (the longest, *trnL1*, in *D. flavistriga*) (Suppl. material 2: table S10). The predicted secondary structures of these tRNAs are shown in Suppl. material 1: figs S1–S4. All 22 tRNAs folded into a typical cloverleaf secondary structure, except for trnS1, which lacked the dihydrouridine (DHU) arm, and instead possessed a single-stranded loop. Wobbled G-U pairs were observed in many tRNA secondary structures; however, they were absent in trnD, trnE, trnH, trnM, trnR, trnS2, and trnY in all four species. Additionally, the rarer U-U pairing was detected, but only in trnA, trnL2 and trnS2 in all four species.

**Figure 4.**
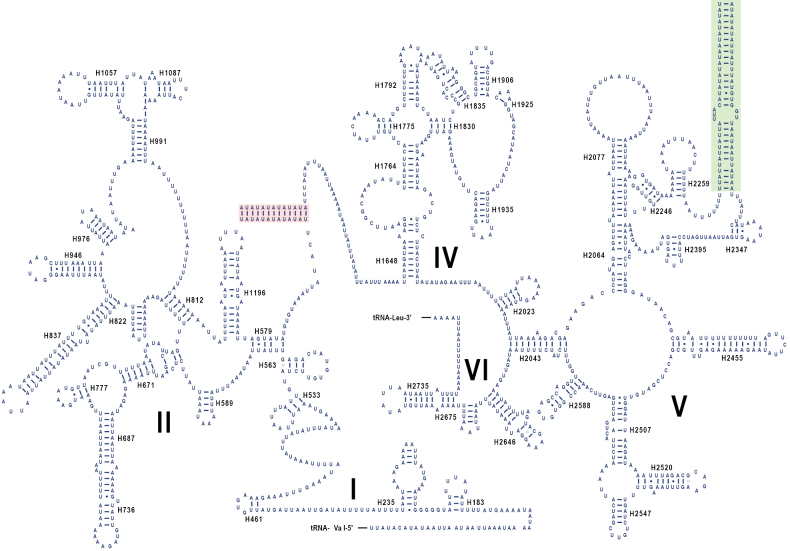
16S rRNA secondary structure of *Warreniplema fumicosta*. The additional helix formed between helices H2259 and H2347 by the microsatellite sequence (TA)_4_A(TA)_7_, along with its short flanking sequences, is highlighted with a pale green background. The additional helix formed between helices H579 and H1648 by the microsatellite sequence (TA)_13_ is highlighted with a pale red background.

The total lengths of the two rRNA genes in the mitogenomes of *D. flavistriga*, *M. prunaria*, *P. alikangensis* and *W. fumicosta* were 2,177 bp, 2,179 bp, 2,195 and 2,237 bp respectively. The 16S rRNA genes were located between *trnL1* and *trnV*, and the 12S rRNA genes were positioned between *trnV* and the control region, with no intergenic spacers or overlaps observed (Suppl. material 2: table S10). The 16S rRNA gene lengths ranged from 1,394 bp (in *D. flavistriga*) to 1,458 bp (in *W. fumicosta*), nearly twice the length of the 12S rRNA gene, which ranged from 779 bp (in *M. prunaria* and *W. fumicosta*) to 783 bp (in *D. flavistriga* and *P. alikangensis*) (Suppl. material 2: table S10). The predicted secondary structures of the two rRNAs for all four species are shown in Fig. 4, Suppl. material 1: figs S5–S11. In the 16S rRNA, five domains (I, II, IV, V, VI) were identified, with domain III absent in Epipleminae, as is typical for all Arthropoda species (Cannone et al. 2002). The 12S rRNA contained three domains (I, II, III). A notable feature shared by all four species was the presence of a (TA)n microsatellite sequence between helices H2259 and H2347 in the 16S rRNA, although the repeat numbers varied: (TA)_21_ in *D. flavistriga*, (TA)_13_ in *M. prunaria*, (TA)_17_ in *P. alikangensis*, and (TA)_4_A(TA)_7_ in *W. fumicosta*. This (TA)n sequence, along with the highly variable short sequences flanking its ends, could form an additional helix in these rRNAs (Fig. 4, Suppl. material 1: figs S5–S7). In addition, another (TA)_13_ repeat was found between helices H579 and H1648 of the 16S rRNA exclusively in *W. fumicosta* (Fig. 4). Such (TA)n microsatellite features in rRNAs are uncommon in insects and have only been reported in tortricid moths (e.g. Gong et al. 2012; Song et al. 2021; Yang et al. 2021).

**Figure 5.**
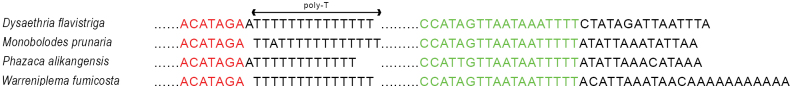
Alignment of the control regions from four newly sequenced epiplemine mitogenomes. The conserved motif ACATAGA (highlighted in red) and its subsequent poly-T stretch, as well as the conserved motif CCATAGTTAATAA(A/T)TTTT (highlighted in green), are emphasized. Dots represent omitted sequences, with the number of dots not proportional to nucleotide number of corresponding region.

The control regions of epiplemine mitogenomes were located between the 12S rRNA and *trnM* genes (Suppl. material 2: table S10). Their lengths in *D. flavistriga*, *M. prunaria*, *P. alikangensis* and *W. fumicosta* were 431 bp, 226 bp, 371 and 411 bp respectively (Table 3). Two conserved blocks potentially associated with transcription or replication, were identified (Fig. 5): An ACATAGA motif followed by a poly-T stretch located downstream of the 12S rRNA gene; a CCATAGTTAATAA(A/T)TTTT motif located upstream of *trnM* gene. One tandem repeat was identified in *W. fumicosta*, with a consensus pattern of TTAATAATAATAATATATTAAATAT (25 bp; copy number: 5.5) and a matching degree of 86%.

### ﻿Phylogenetic analyses

**Figure 6.**
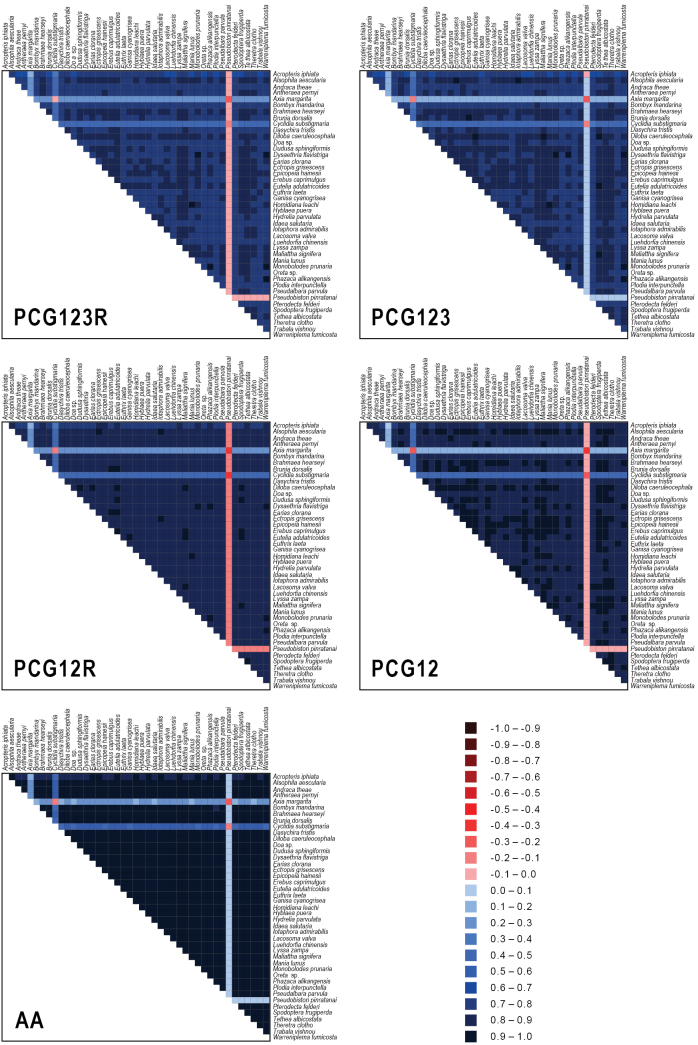
Base compositional heterogeneity of mitogenome sequences for our five nucleotide and amino acid datasets used in phylogenetic analyses. The degree of sequence similarity is visualized using colored blocks, based on AliGROOVE scores, ranging from -1 (indicating non-random similarity, shown in red) to +1 (representing full random similarity, shown in blue).

The AliGROOVE tests revealed generally low sequence composition heterogeneity among the 43 representative species across all five datasets, with the exception of three species: *Axia margarita* (Cimeliidae), *Cyclidia substigmaria* (Cyclidiinae) and *Pseudobiston pinratanai* (Pseudobistonidae) (Fig. 6). The higher heterogeneity observed in these three species was primarily attributed to the incomplete assembly of their mitogenomic sequences. Notably, due to the higher mutation rate of the third codon position in protein-coding genes, the PCG123R and PCG123 datasets exhibited higher heterogeneity than the PCG12R and PCG12 datasets, where the third codon positions were excluded. The least heterogeneous dataset was the AA set, a result of codon degeneracy.

**Figure 7.**
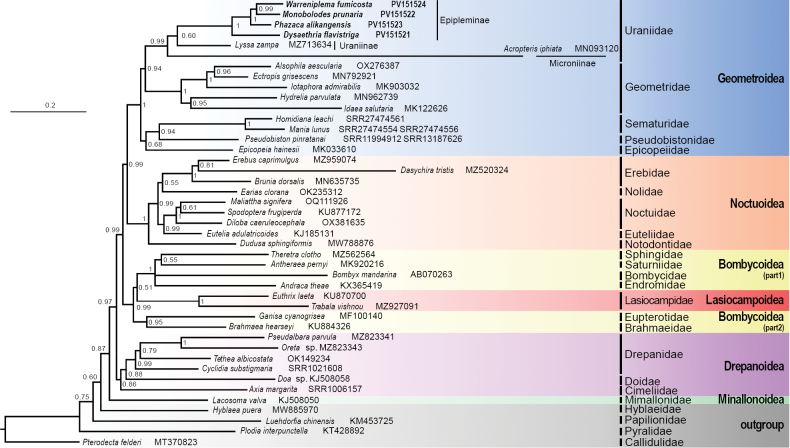
Phylogenetic tree of Macroheterocera produced by Bayesian Inference (BI) based on the AA dataset. Numerals at nodes are Bayesian posterior probabilities (PP).

Different combinations of datasets, partitioning models, and tree-building methods resulted in ten phylogenetic trees within Macroheterocera (Fig. 7; Suppl. material 1: figs S12–S20). The six superfamilies (Bombycoidea, Drepanoidea, Geometroidea, Lasiocampoidea, Mimallonoidea, and Noctuoidea) of Macroheterocera were successfully clustered into one monophyletic clade in nine out of the ten trees (BS: 85–92, PP: 0.87–1.00; Fig. 7; Suppl. material 1: figs S12–S19). The relationships recovered in most analyses were as follows: Mimallonoidea + (Drepanoidea + ((Bombycoidea + Lasiocampoidea) + (Noctuoidea + Geometroidea))). This topology is almost identical with that of a previous phylogenetic study also based on mitogenomic data (Yang et al. 2019b). The clade (Bombycoidea + Lasiocampoidea) + (Noctuoidea + Geometroidea) was robustly recovered in all ten trees (BS: 89–97, PP: 0.99–1.00). However, the position of Mimallonoidea, which includes only a single family (Mimallonidae), was unstable across our trees. In the ML tree built with the AA dataset, Mimallonoidea formed a novel branch with a species from the outgroup Hyblaeidae (BS: 72; Suppl. material 1: fig. S20), rendering Macroheterocera paraphyletic. In the BI trees produced with the PCG123R and PCG123 datasets, Mimallonoidea clustered with Cimeliidae (belonging to Drepanoidea) (PP: 0.88, 0.66; Suppl. material 1: figs S13, S15) as a sister group to the rest of Macroheterocera, which included the remaining Drepanoidea, thereby rendering Drepanoidea polyphyletic. In the ML tree produced with the PCG12 dataset, Mimallonoidea was positioned as sister to the (Bombycoidea + Lasiocampoidea) + (Noctuoidea + Geometroidea) clade (Suppl. material 1: fig. S18), with weak nodal support (BS: 60). In all other analyses, Mimallonoidea was placed at the base of Macroheterocera, consistent with most previous studies (e.g. Kawahara et al. 2019; Yang et al. 2019b; Mayer et al. 2021; Rota et al. 2022).

Drepanoidea (comprising three families: Cimeliidae, Doidae, and Drepanidae sensu van Nieukerken et al. (2011)) was recovered as a monophyletic lineage sister to (Bombycoidea + Lasiocampoidea) + (Noctuoidea + Geometroidea) in most of our analyses (BS: 53–84, PP: 0.95–0.98; Figs 7, Suppl. material 1: figs S12, S14, S16, S17, S19, S20). This position is supported in most recent phylogenetic studies (Bazinet et al. 2013; Timmermans et al. 2014; Kawahara et al. 2019; Yang et al. 2019b; Rota et al. 2022; Mayer et al. 2021). Within Drepanoidea, Drepanidae was consistently recovered as sister to Doidae (BS: 83–99, PP: 0.88–1.00), and these in turn sister to Cimeliidae. However, in two BI analyses produced with the PCG123R and PCG123 datasets, Cimeliidae branched into the same clade with Mimallonoidea (PP: 0.88, 0.66; Suppl. material 1: figs S13, S15).

The close relationship between Bombycoidea and Lasiocampoidea was strongly supported by all our analyses (BS: 99–100, PP: 0.99–1.00). However, their sibling relationship, as suggested by multiple previous studies (e.g. Kawahara et al. 2019; Mayer et al. 2021; Rota et al. 2022), was only recovered in one of our analyses (the ML tree with the PCG12R dataset; Suppl. material 1: fig. S16). In all other analyses, Lasiocampoidea was consistently nested within Bombycoidea, rendering the latter paraphyletic. This unconventional topology has also been reported in two phylomitogenomic studies (Xin et al. 2017; Yang et al. 2019b), further highlighting the contentious nature of the relationship between Bombycoidea and Lasiocampoidea.

The relationship between Geometroidea and Noctuoidea has historically been unresolved, despite a number of molecular phylogenetic studies addressing this issue (e.g. Yang et al. 2019b; Mayer et al. 2021; Rota et al. 2022). In our study, however, Geometroidea and Noctuoidea were recovered as sister groups in all analyses, with very strong nodal support (BS: 95–100, PP: 0.97–1.00). This result aligns with several previous phylogenetic studies (Kawahara and Breinholt 2014; Yang et al. 2019b; Cheng et al. 2022).

The superfamily Geometroidea was consistently recovered as monophyletic in all our analyses, with very strong nodal support (BS: 98–100, PP: 1.00). All the five families (Epicopeiidae, Geometridae, Pseudobistonidae, Sematuridae, and Uraniidae) included in Geometroidea sensu Mitter et al. (2017) were resolved into three major lineages: The first comprising Geometridae (BS: 100, PP: 1.00); the second comprising Uraniidae (BS: 98–100, PP: 0.99–1.00); the third comprising Epicopeiidae + (Pseudobistonidae + Sematuridae) (BS: 73–88, PP: 0.68–0.83). However, the relationships among these three lineages remained unresolved in our study. Their relative placements in our trees were unstable and conflicting, although most recent phylogenetic studies have favored a sister relationship between Geometridae and Uraniidae (Bazinet et al. 2013; Regier et al. 2013; Rajaei et al. 2015; Kawahara et al. 2019; Murillo-Ramos et al. 2019; Wang et al. 2019; Mayer et al. 2021). Within the third lineage, Pseudobistonidae was recovered closest to Sematuridae (BS: 69–96, PP: 0.94–0.99), rather than the commonly supported Epicopeiidae (Rajaei et al. 2015; Wang et al. 2019), reinforcing the result of the latest phylogenomic study on these groups (Mayer et al. 2021). All four species sequenced in our study formed a highly supported monophyletic clade representing the subfamily Epipleminae (BS: 100, PP: 1.00). Furthermore, Epipleminae, Microniinae and Uraniinae successfully clustered into the monophyletic family Uraniidae (BS: 98–100, PP: 0.99–1.00).

## ﻿Conclusions

In this study, we presented the first four mitogenomes of the subfamily Epipleminae, providing detailed comparative analyses and exploring their phylogenetic implications within Macroheterocera. While the organization and gene content of these mitogenomes did not exhibit significant divergence from other ditrysian Lepidoptera, a rare phenomenon within Lepidoptera–previously reported only in tortricid mitogenomes–was observed in the 16S rRNA, where additional helices were formed by (TA)n microsatellite sequences in all four species. Specifically, one helix, located between helices H2259 and H2347, was conserved across all four species, although its sequence composition varied among them. Additionally, a unique (TA)_13_ helix was identified exclusively in *W. fumicosta*, positioned between helices H579 and H1648. Noteworthily, these additional helices were not observed in the three previously published mitogenome sequences (GenBank Nos MN093120, MW435592, and MZ713634) from the other two subfamilies of Uraniidae.

In our phylogenetic analyses, the generated trees were largely congruent with previous molecular studies regarding the major lineages within Macroheterocera on the superfamily level. Our study was the first mitogenomic-based analysis to include representatives from all five families within the superfamily Geometroidea. The monophyly of Geometroidea and its sister relationship with Noctuoidea were strongly supported. All four species sequenced in this study formed a highly supported monophyletic clade, representing the subfamily Epipleminae. Furthermore, representatives from Epipleminae, Microniinae, and Uraniinae clustered into the monophyletic family Uraniidae within Geometroidea. However, due to the absence of mitogenomic data from the subfamily Auzeinae, and the overall scarcity of representative mitogenomes from Uraniidae, the monophyly of Uraniidae could not be fully tested in our study, and the internal phylogenetic relationships within Uraniidae remained unresolved. These findings highlighted the critical need for additional mitogenomic data from underrepresented taxa to further refine the phylogenetic framework of Uraniidae and to elucidate the evolutionary relationships within this diverse family.

## ﻿Additional information

### Use of AI

No use of AI was reported.

### Author ORCIDs

Yanpeng Cai https://orcid.org/0000-0001-6554-3784

Aihui Yin https://orcid.org/0000-0001-7614-9969

### Data availability

Data presented in this study are openly available in the GenBank repository under accession numbers: PV151521–PV151524 and in the BOLD system with sample IDs GZUTCM:SWD, GZUTCM:SWM, GZUTCM:SWP, and GZUTCM:SWW.

## Supplementary materials

Supplementary material 1 Supplementary figures Data type pdf Explanation note **figs S1–S4.** tRNA secondary structures of *Dysaethria flavistriga*, *Monobolodes prunaria*, *Phazaca alikangensis*, and *Warreniplema fumicosta*. **figs S5–S7.** 16S rRNA secondary structures of *Dysaethria flavistriga*, *Monobolodes prunaria*, and *Phazaca alikangensis*. **figs S8–S11.** 12S rRNA secondary structures of *Dysaethria flavistriga*, *Monobolodes prunaria*, *Phazaca alikangensis*, and *Warreniplema fumicosta*. **figs S12–S20.** Phylogenetic trees of Macroheterocera. https://binary.pensoft.net/file/1440257This dataset is made available under the Open Database License (http://opendatacommons.org/licenses/odbl/1.0/). The Open Database License (ODbL) is a license agreement intended to allow users to freely share, modify, and use this Dataset while maintaining this same freedom for others, provided that the original source and author(s) are credited.Yanpeng Cai, Aihui Yin

Supplementary material 2 Supplementary tables Data type pdf Explanation note **tables S1–S9.** The corresponding partitioning schemes and substitution models determined by ModelFinder for the phylogenetic analyses using site-homogeneous models. **table S10.** Annotation of the mitochondrial genomes of four epiplemine species. https://binary.pensoft.net/file/1440258This dataset is made available under the Open Database License (http://opendatacommons.org/licenses/odbl/1.0/). The Open Database License (ODbL) is a license agreement intended to allow users to freely share, modify, and use this Dataset while maintaining this same freedom for others, provided that the original source and author(s) are credited.Yanpeng Cai, Aihui Yin
