# Supplementary material for: ﻿The first mitogenomes of the subfamily Epipleminae (Lepidoptera, Uraniidae) and phylogenetic analysis of Macroheterocera
Source: Zookeys. 2025 Oct 15;1255:343–63. doi: 10.3897/zookeys.1255.164711 (PMC12547423; doi:10.3897/zookeys.1255.164711)
Supplement: Supplementary material 1 — Supplementary figures [file zookeys-1255-343_article-164711__-s001.pdf]

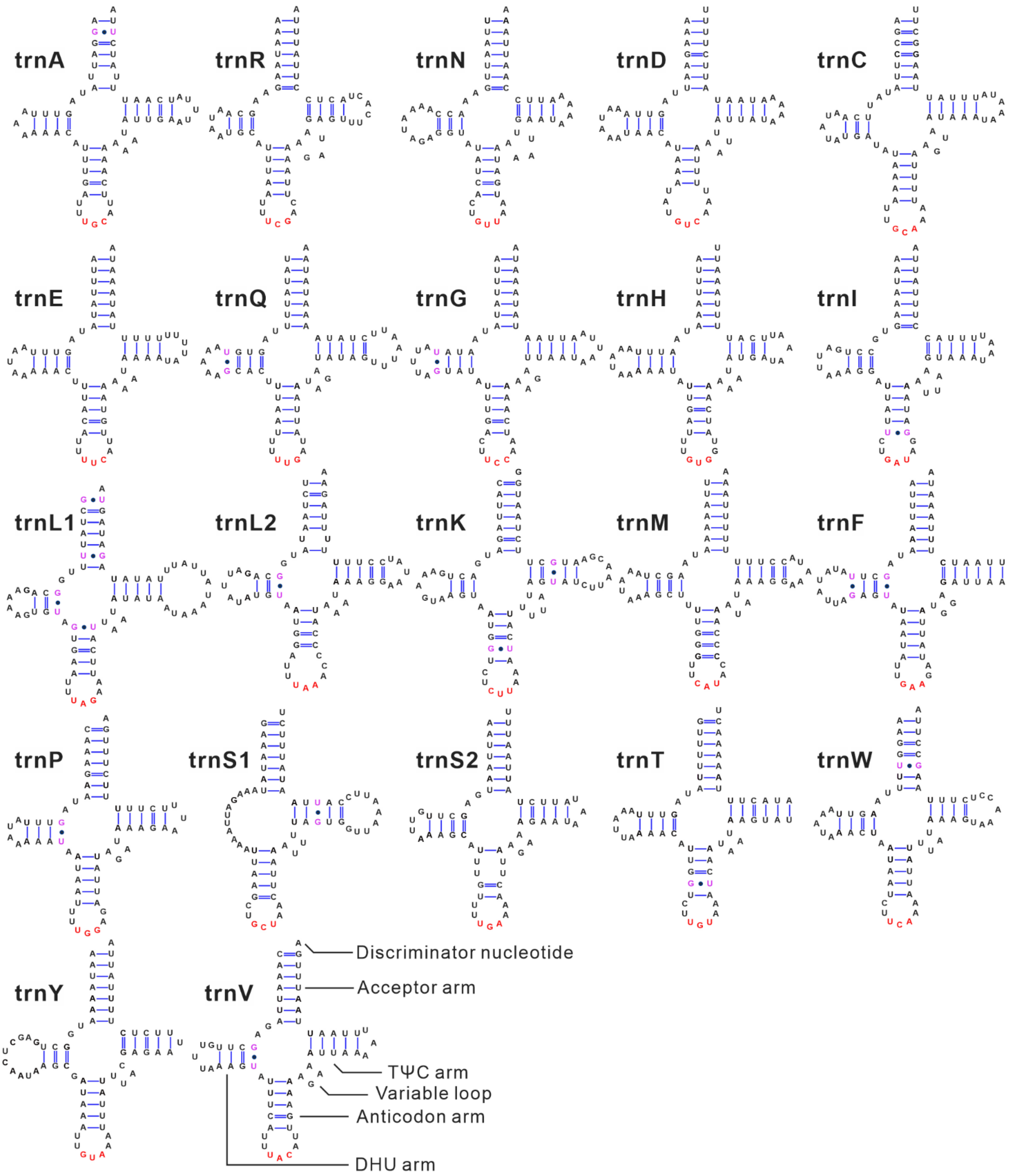

Figure S1. tRNA secondary structures of *Dysaethria flavistriga*.

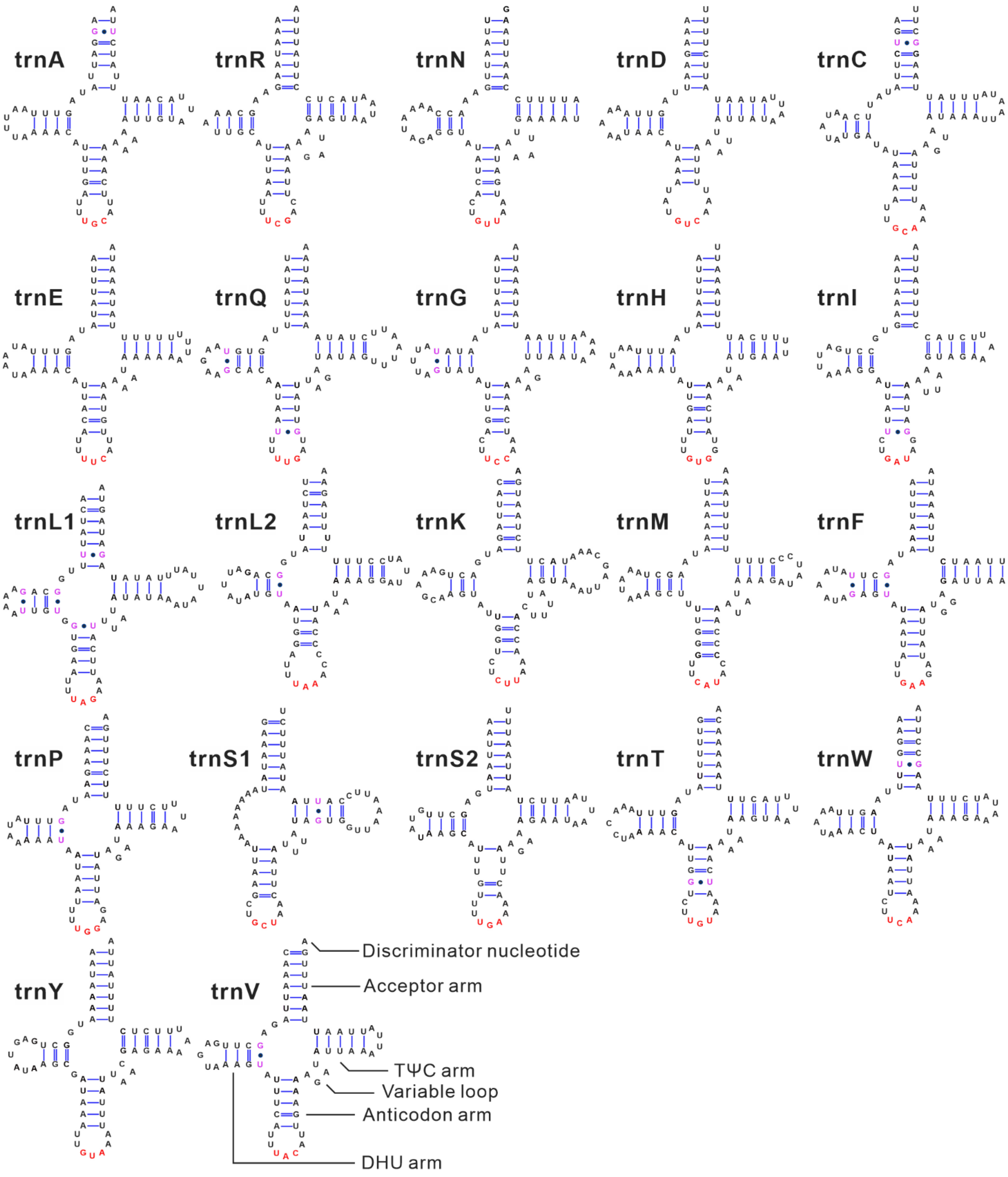

Figure S2. tRNA secondary structures of *Monobolodes prunaria*.

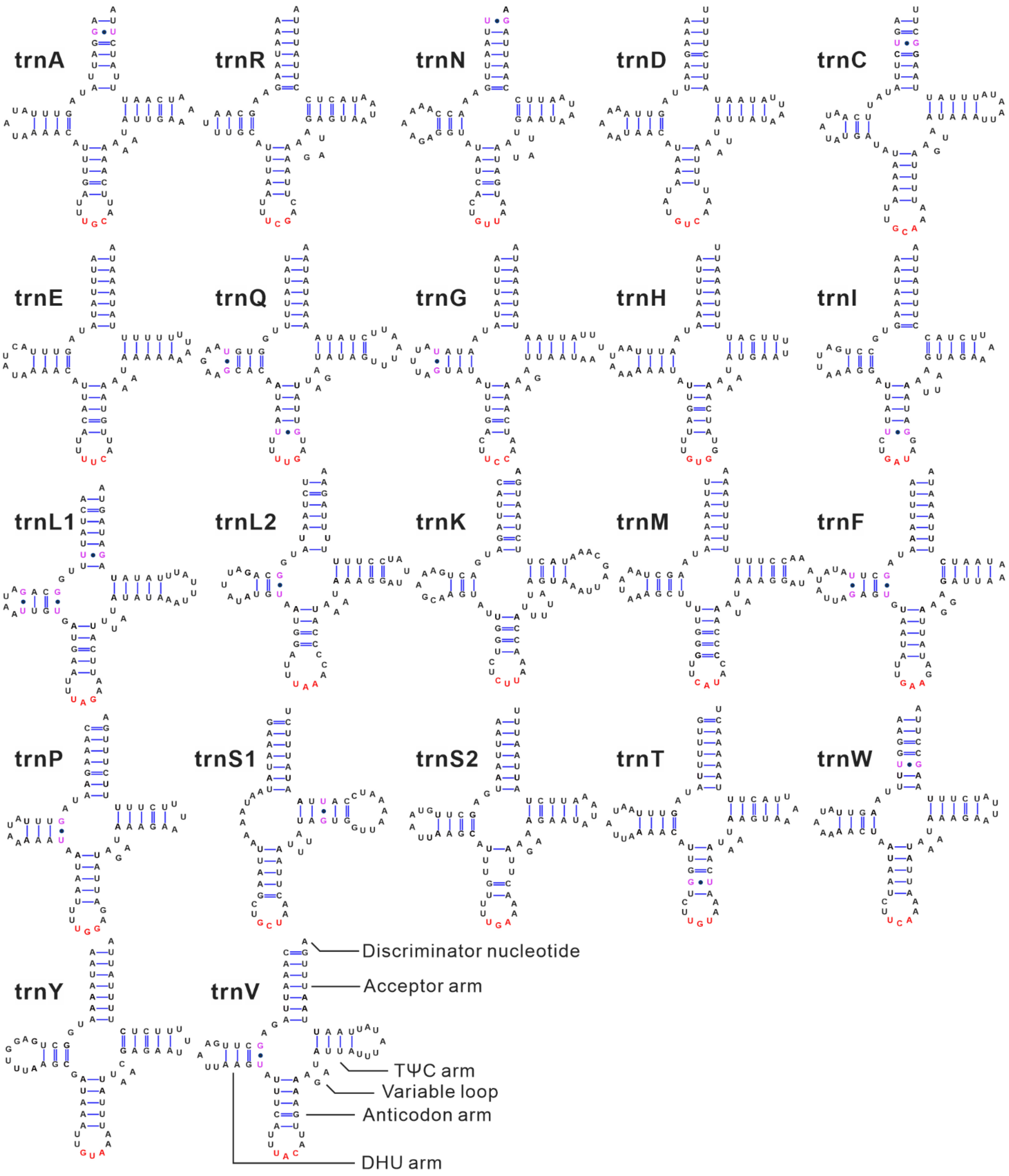

Figure S3. tRNA secondary structures of *Phazaca alikangensis*.

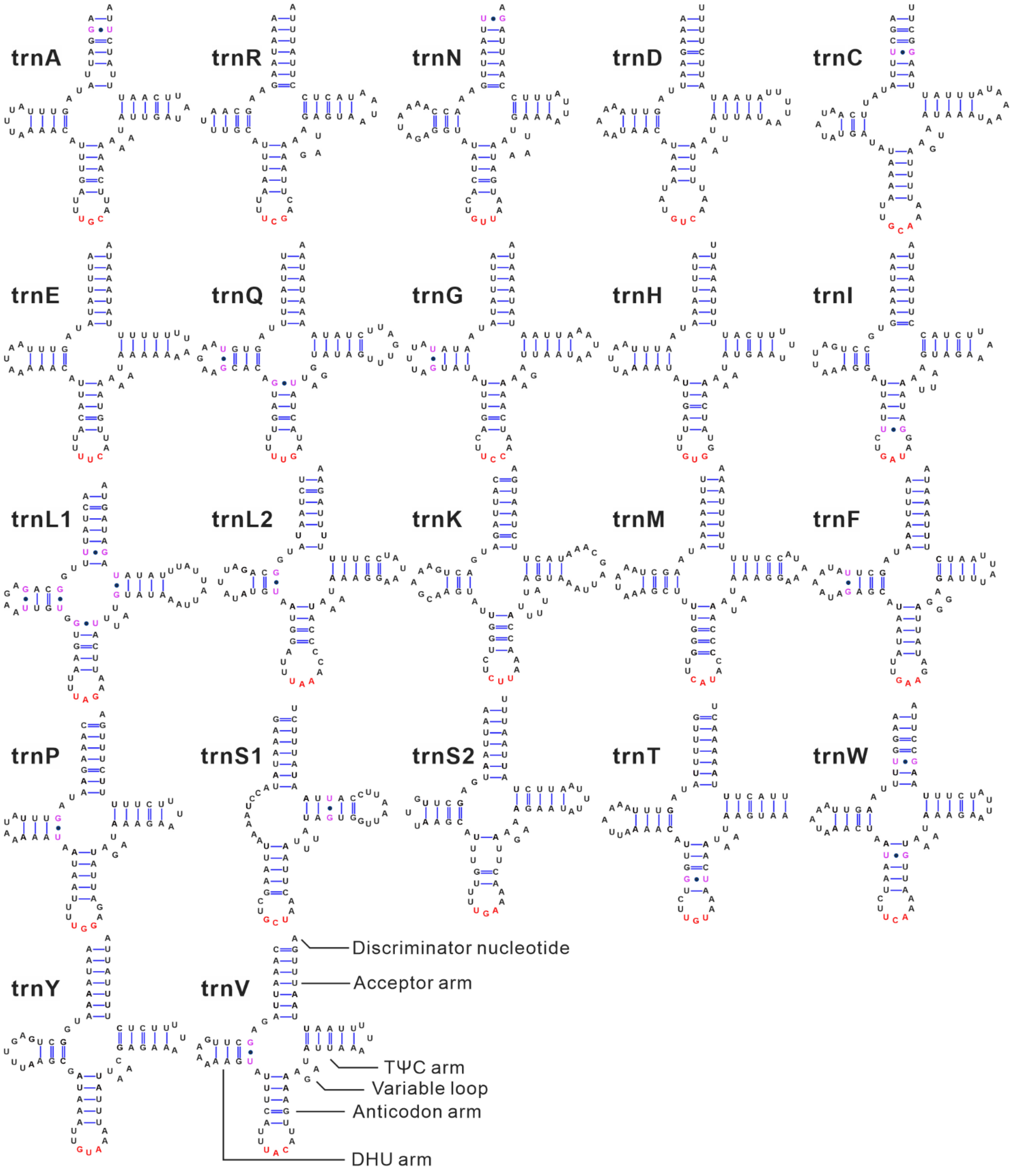

Figure S4. tRNA secondary structures of *Warreniplema fumicosta*.

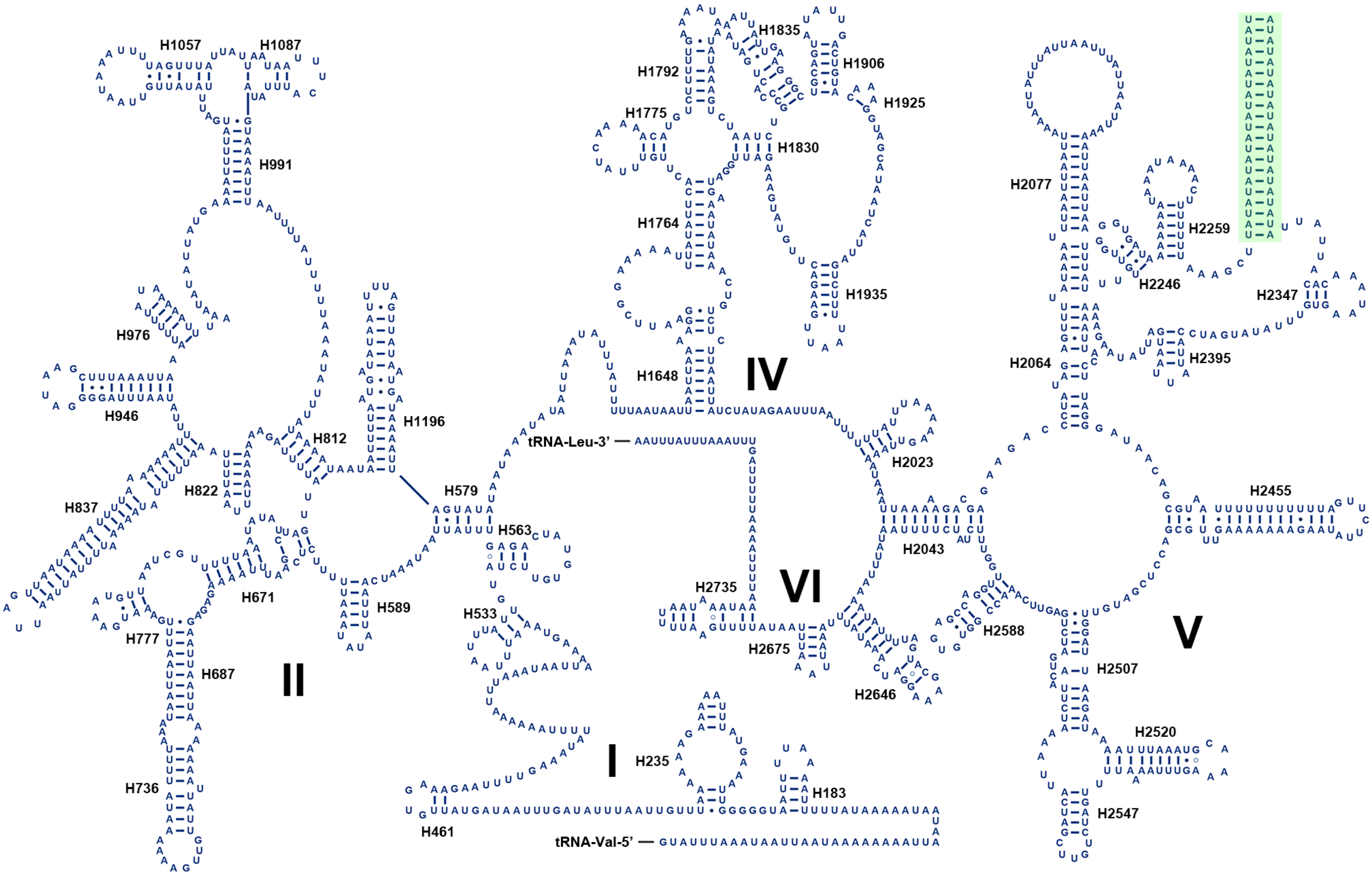

Figure S5. 16S rRNA secondary structure of *Dysaethria flavistriga*. The additional helix formed between helices H2259 and H2347 by the microsatellite sequence $(TA)_{21}$, along with its short flanking sequences, is highlighted with a light green background.

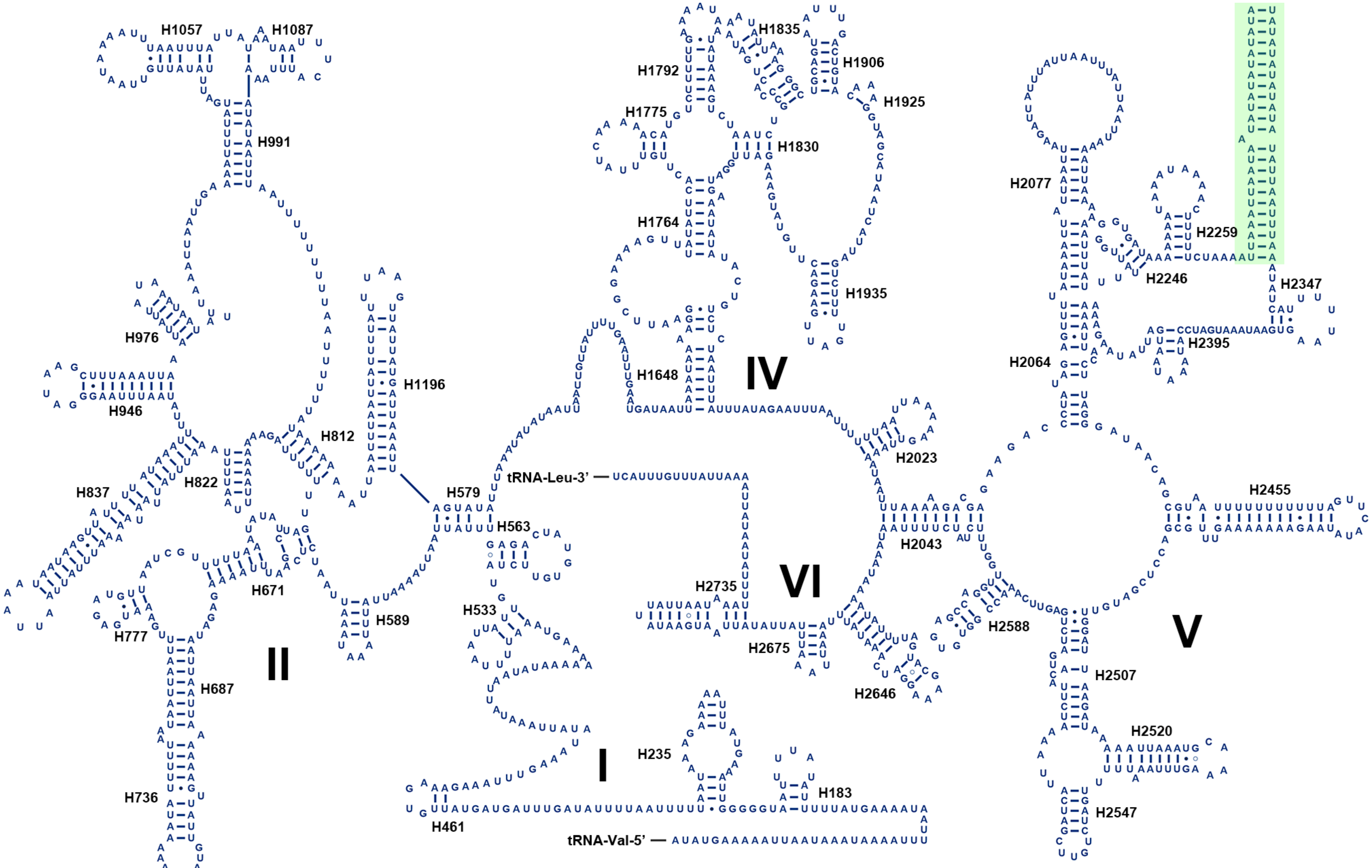

Figure S6. 16S rRNA secondary structure of *Monobolodes prunaria*. The additional helix formed between helices H2259 and H2347 by the microsatellite sequence $(TA)_{13}$, along with its short flanking sequences, is highlighted with a light green background.

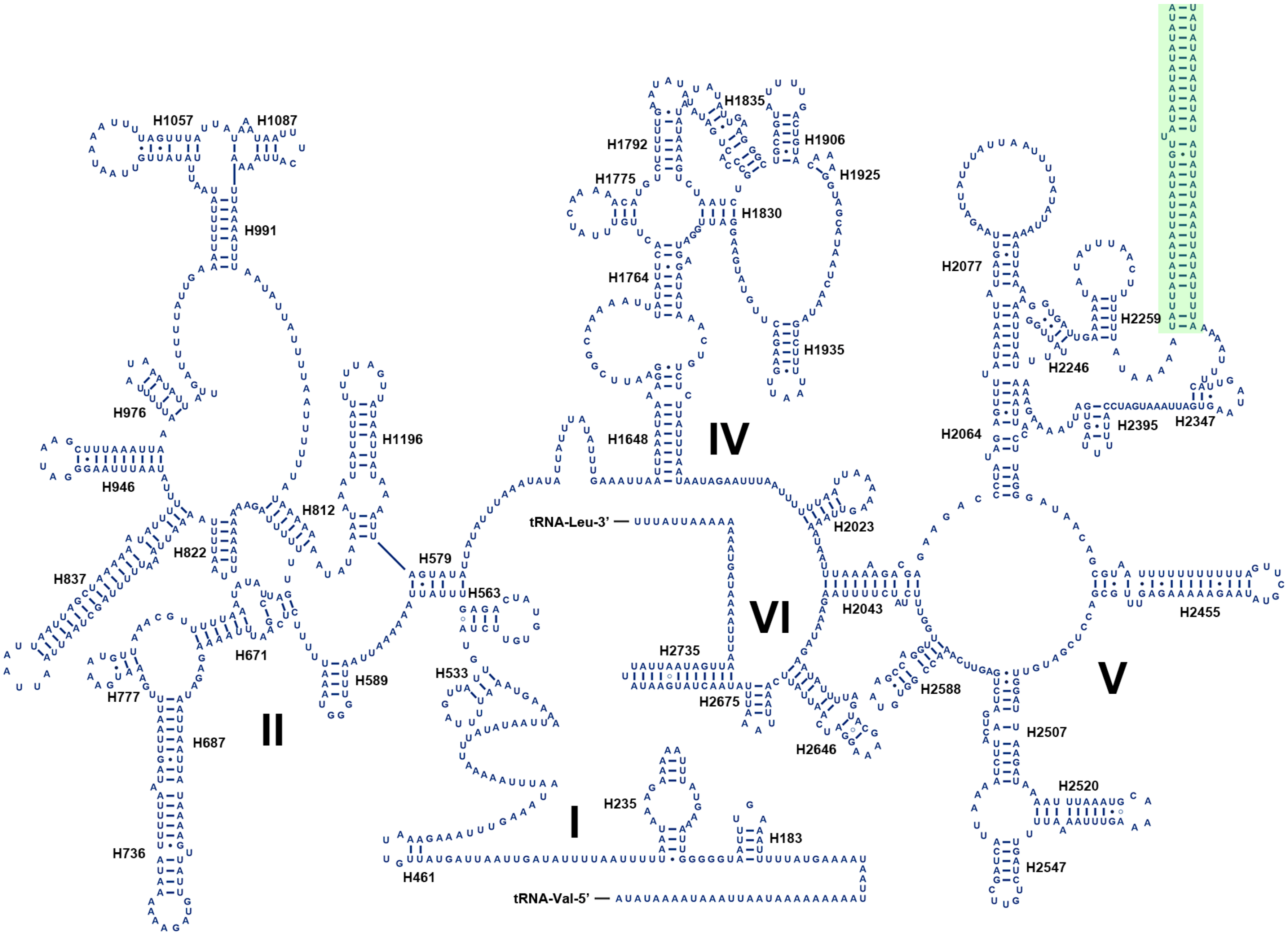

Figure S7. 16S rRNA secondary structure of *Phazaca alikangensis*. The additional helix formed between helices H2259 and H2347 by the microsatellite sequence $(TA)_{17}$, along with its short flanking sequences, is highlighted with a light green background.

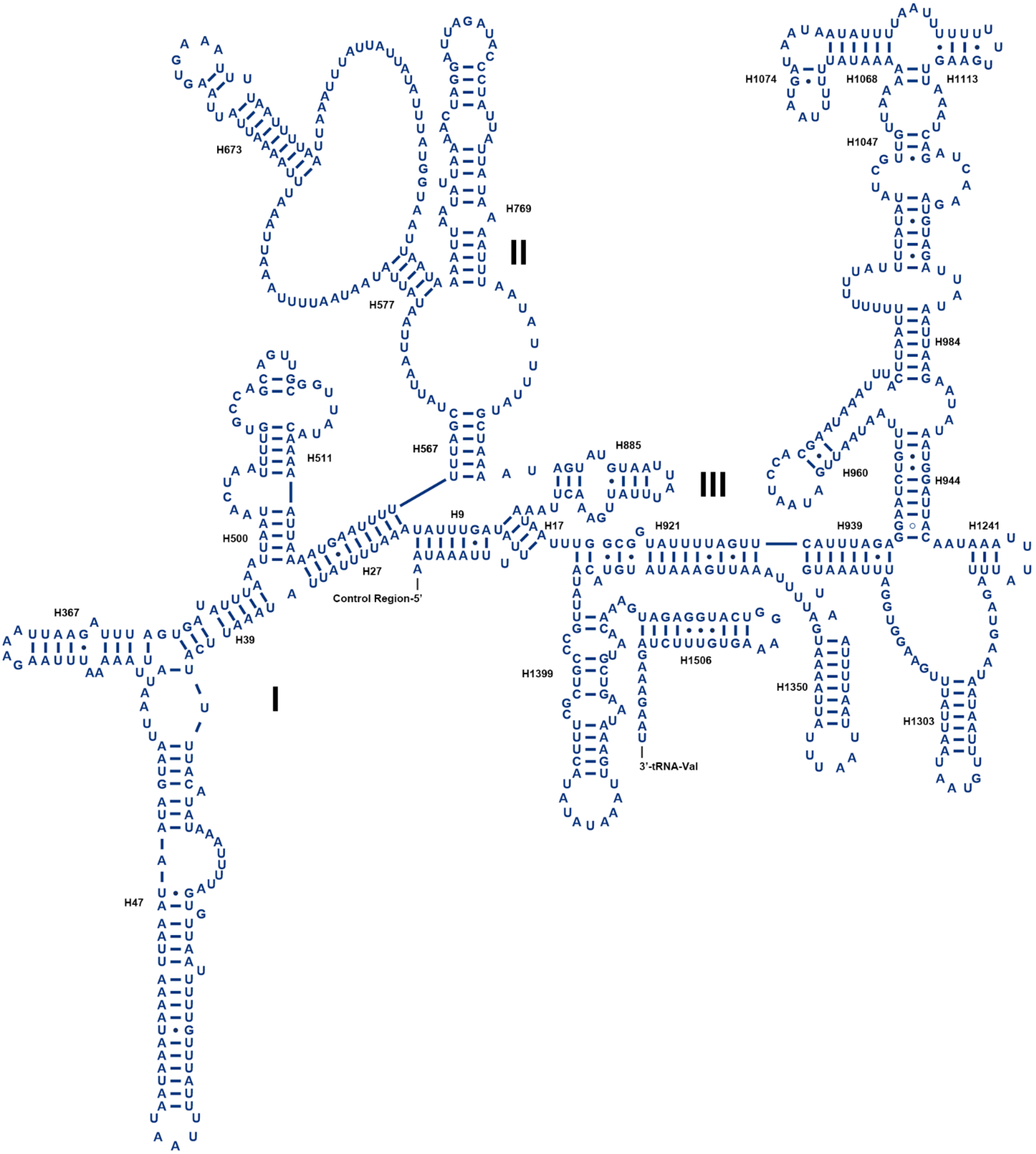

Figure S8. 12S rRNA secondary structure of *Dysaethria flavistriga*.

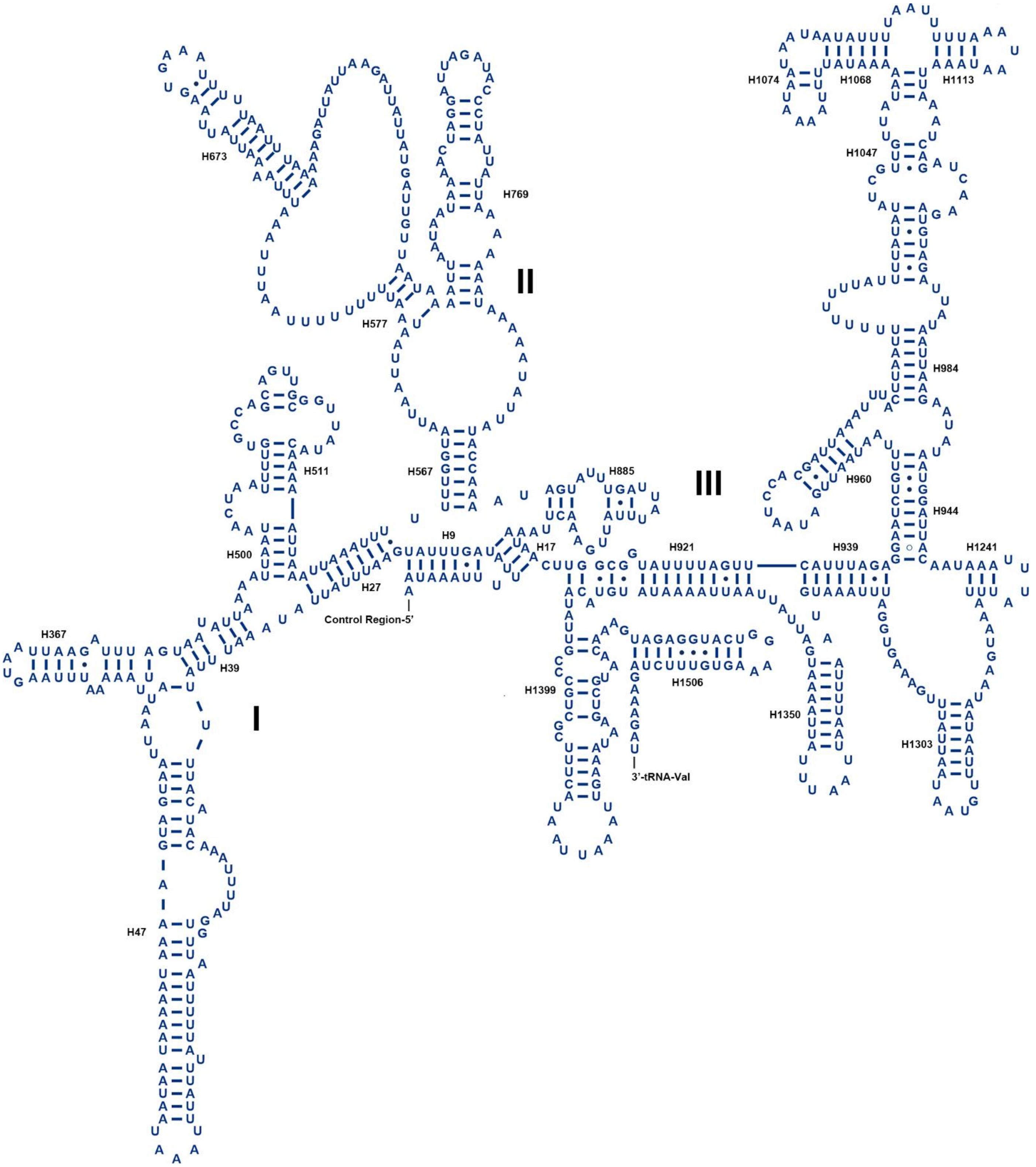

Figure S9. 12S rRNA secondary structure of *Monobolodes prunaria*.

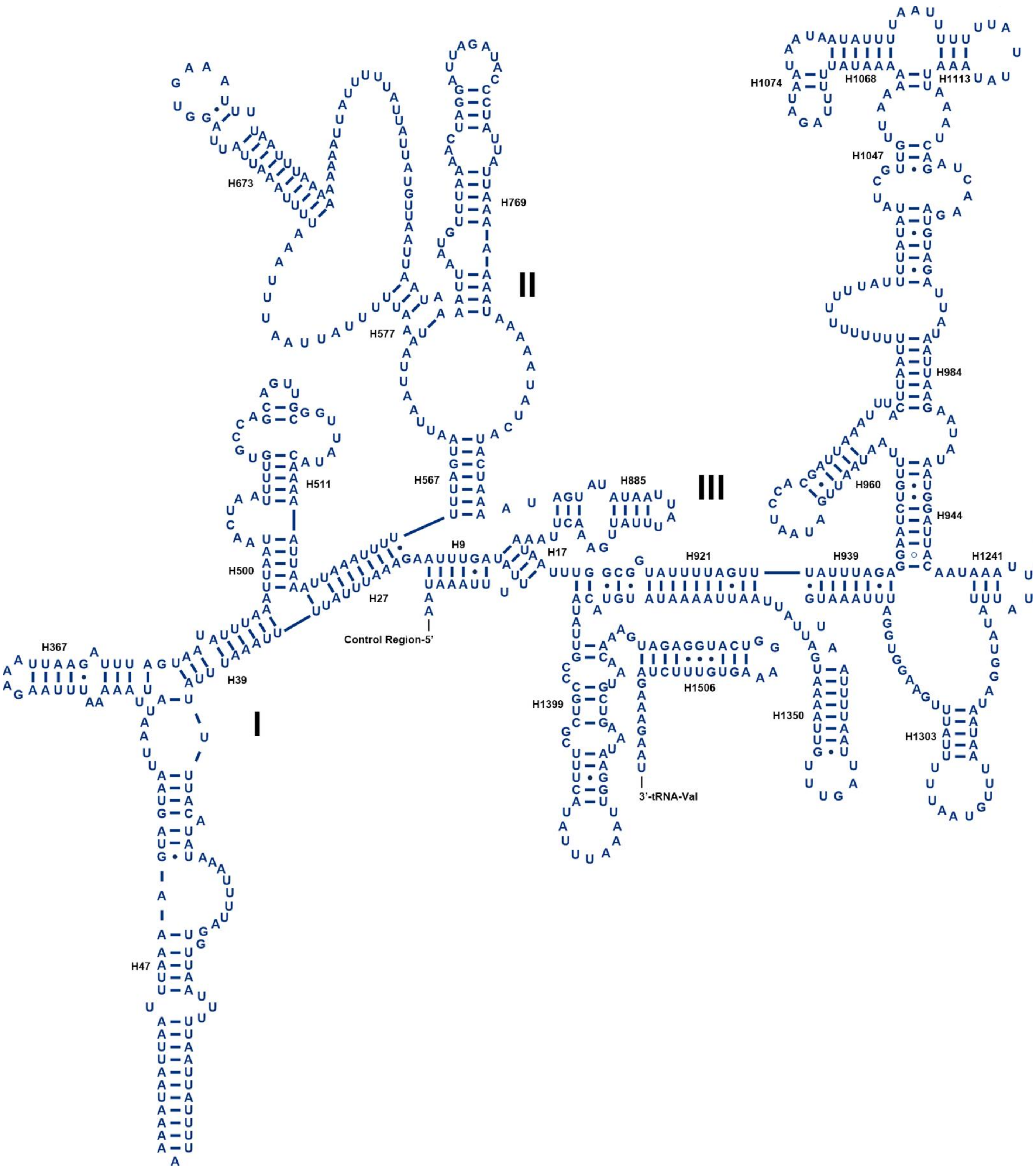

Figure S10. 12S rRNA secondary structure of *Phazaca alikangensis*.

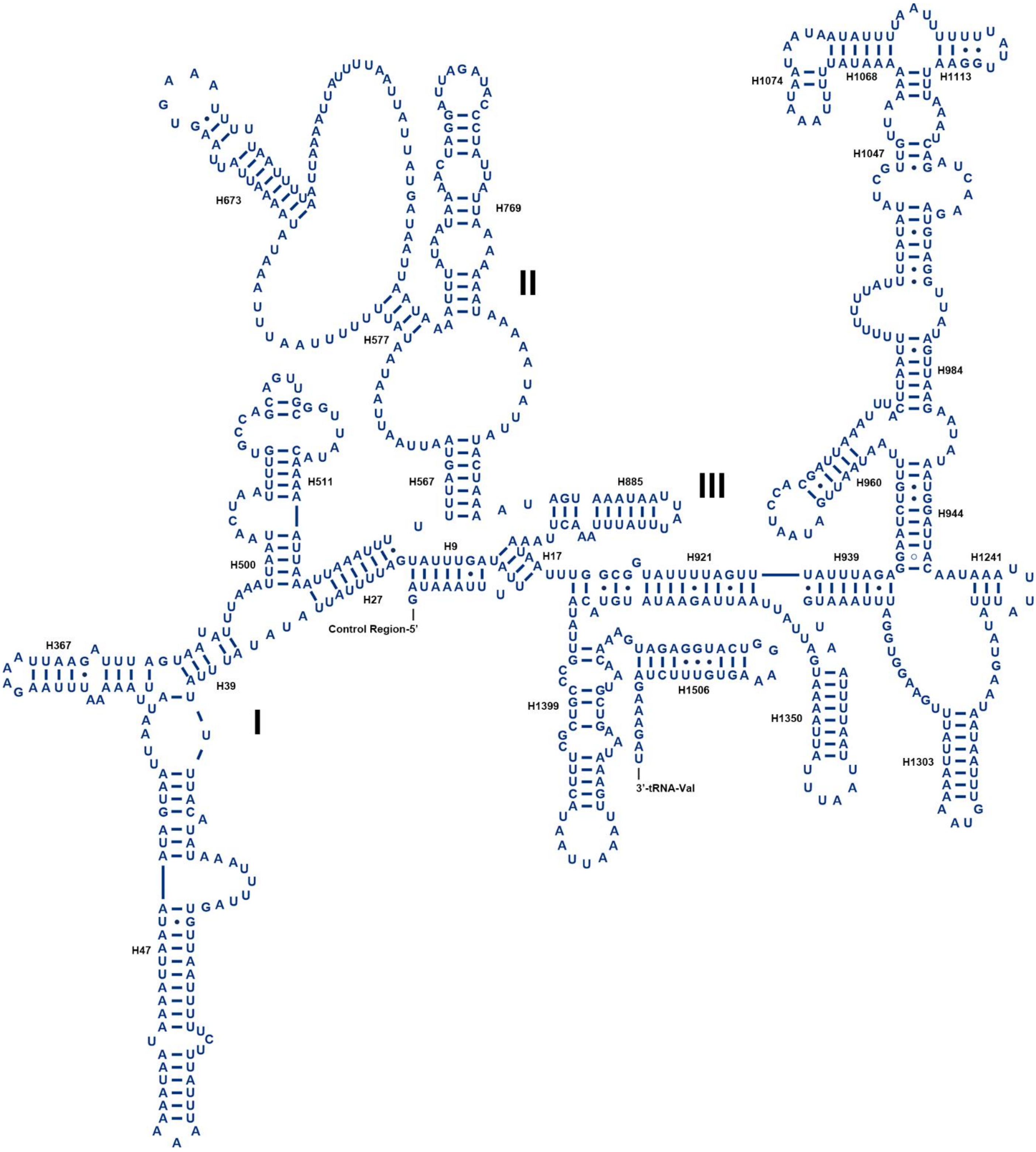

Figure S11. 12S rRNA secondary structure of *Warreniplema fumicosta*.

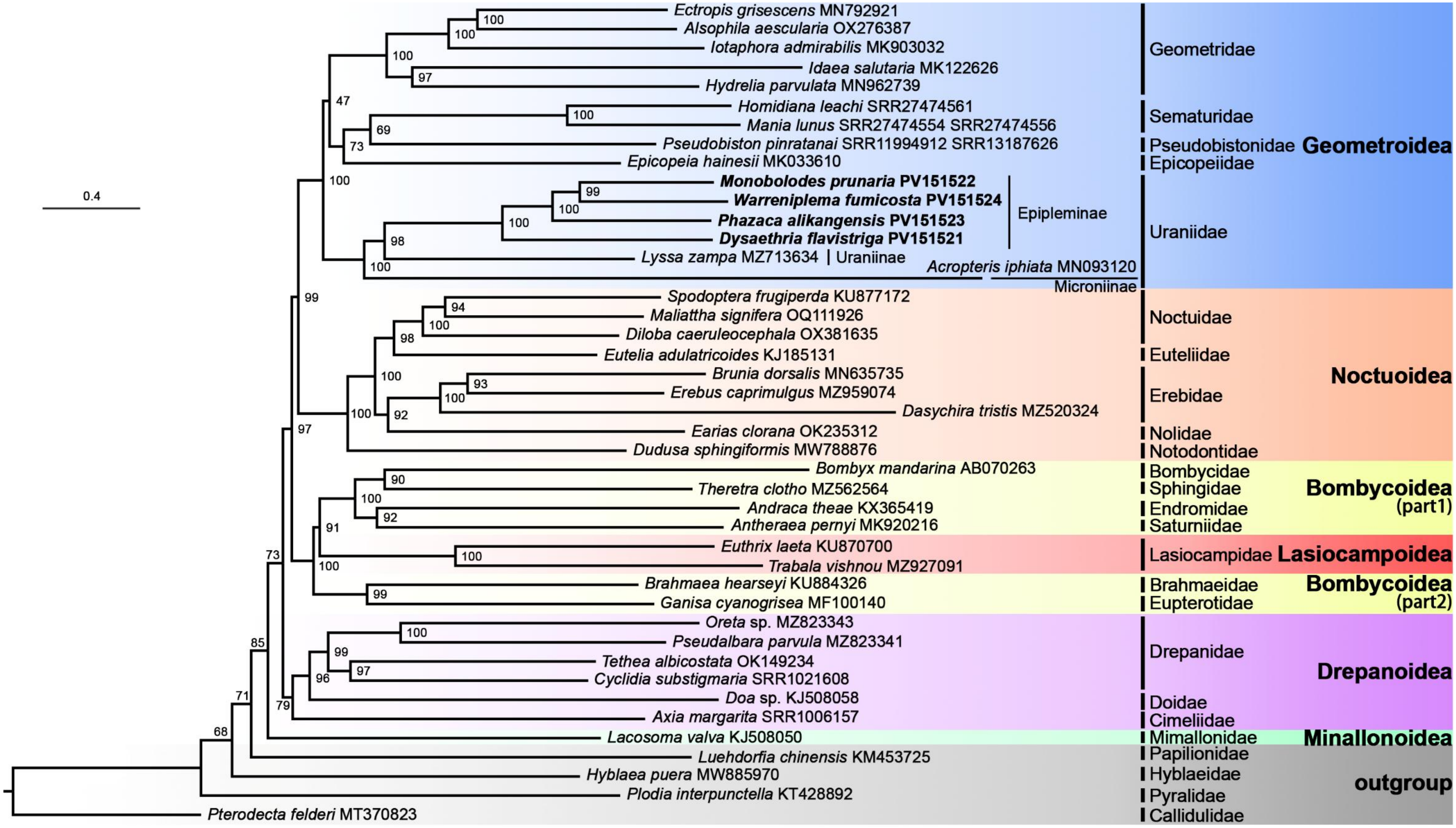

Figure S12. Phylogenetic tree of Macroheterocera produced by Maximum Likelihood (ML) based on the PCG123R dataset. Numerals at nodes are bootstrap support values (BS).

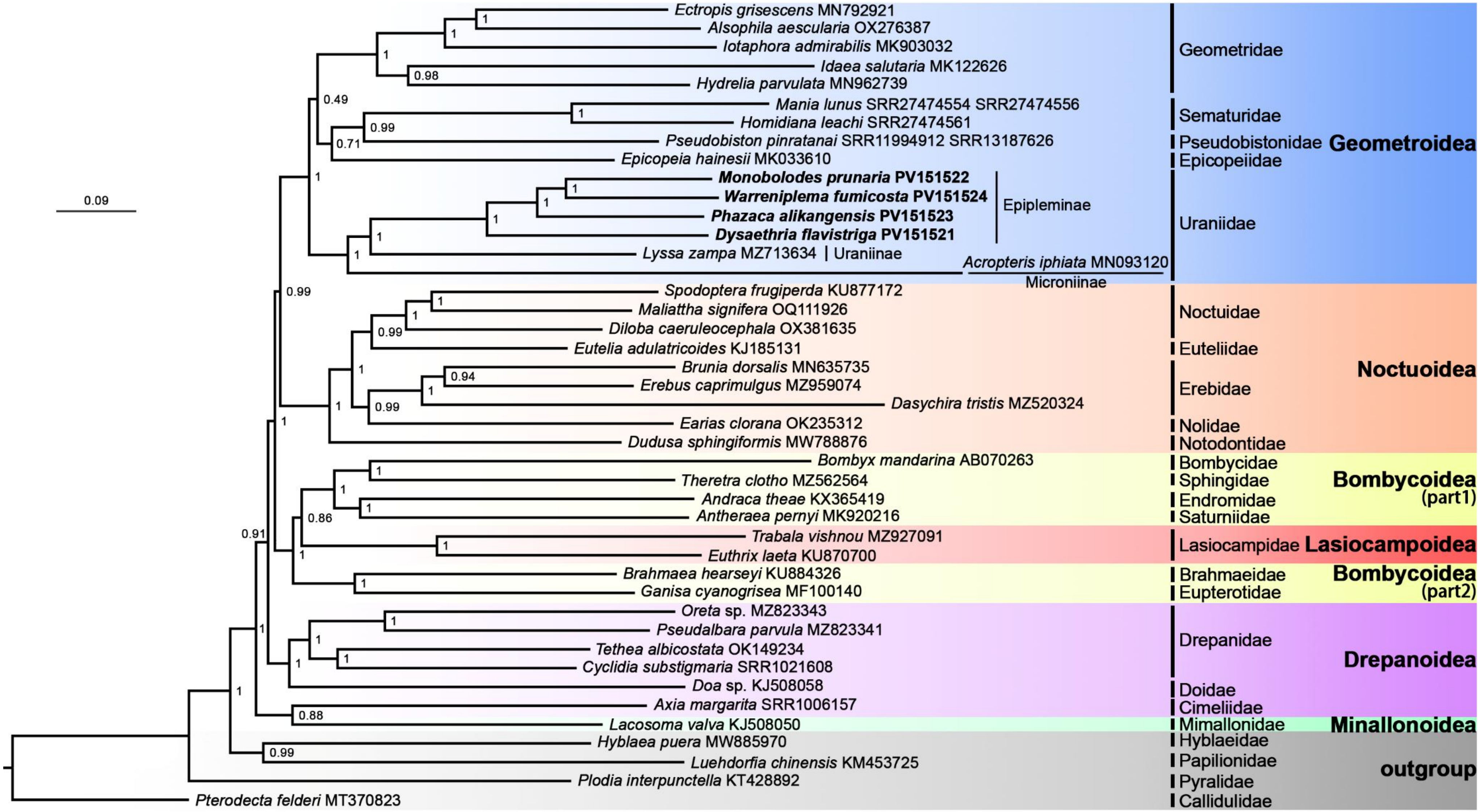

Figure S13. Phylogenetic tree of Macroheterocera produced by Bayesian Inference (BI) based on the PCG123R dataset. Numerals at nodes are Bayesian posterior probabilities (PP).

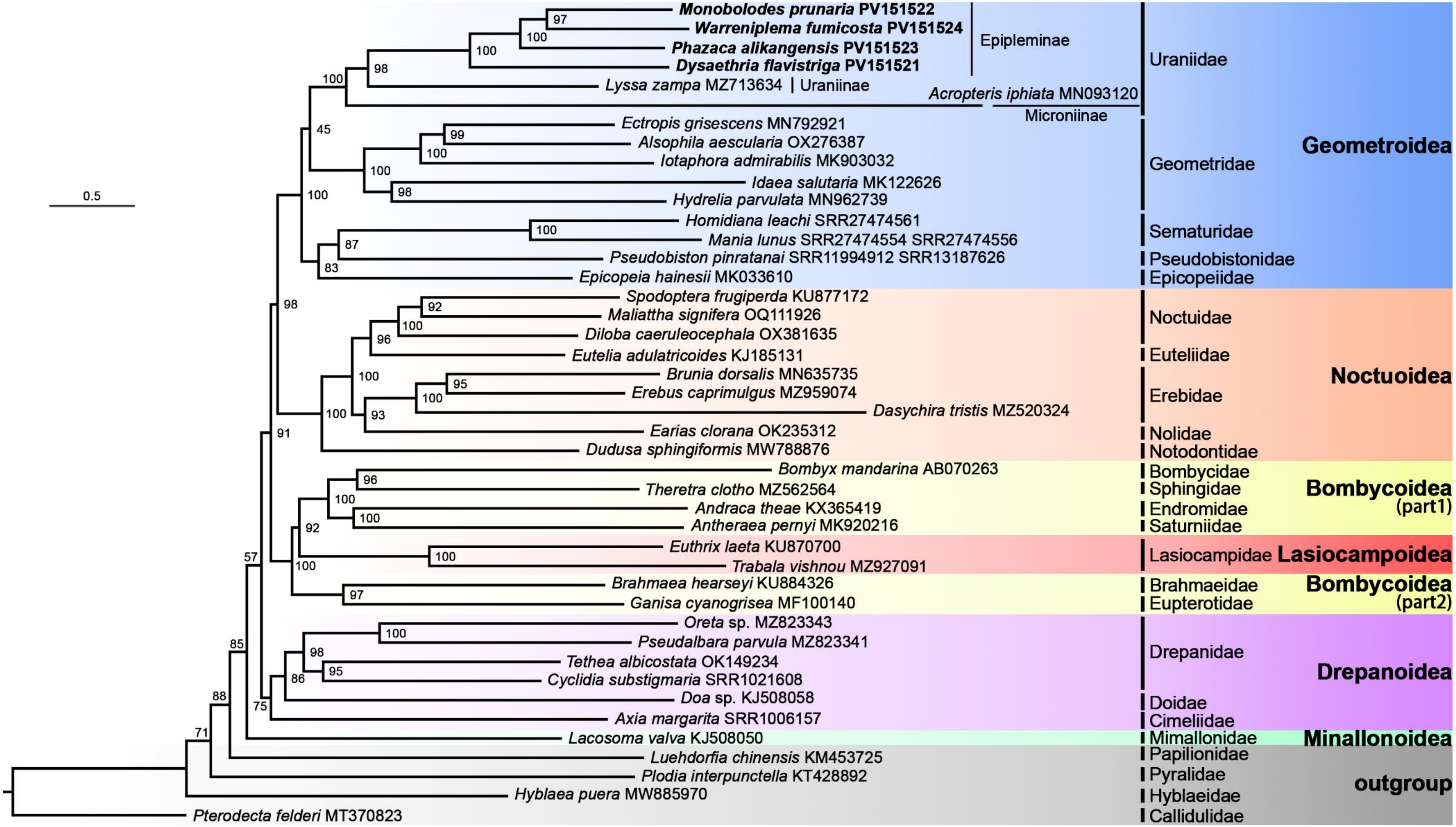

Figure S14. Phylogenetic tree of Macroheterocera produced by Maximum Likelihood (ML) based on the PCG123 dataset. Numerals at nodes are bootstrap support values (BS).

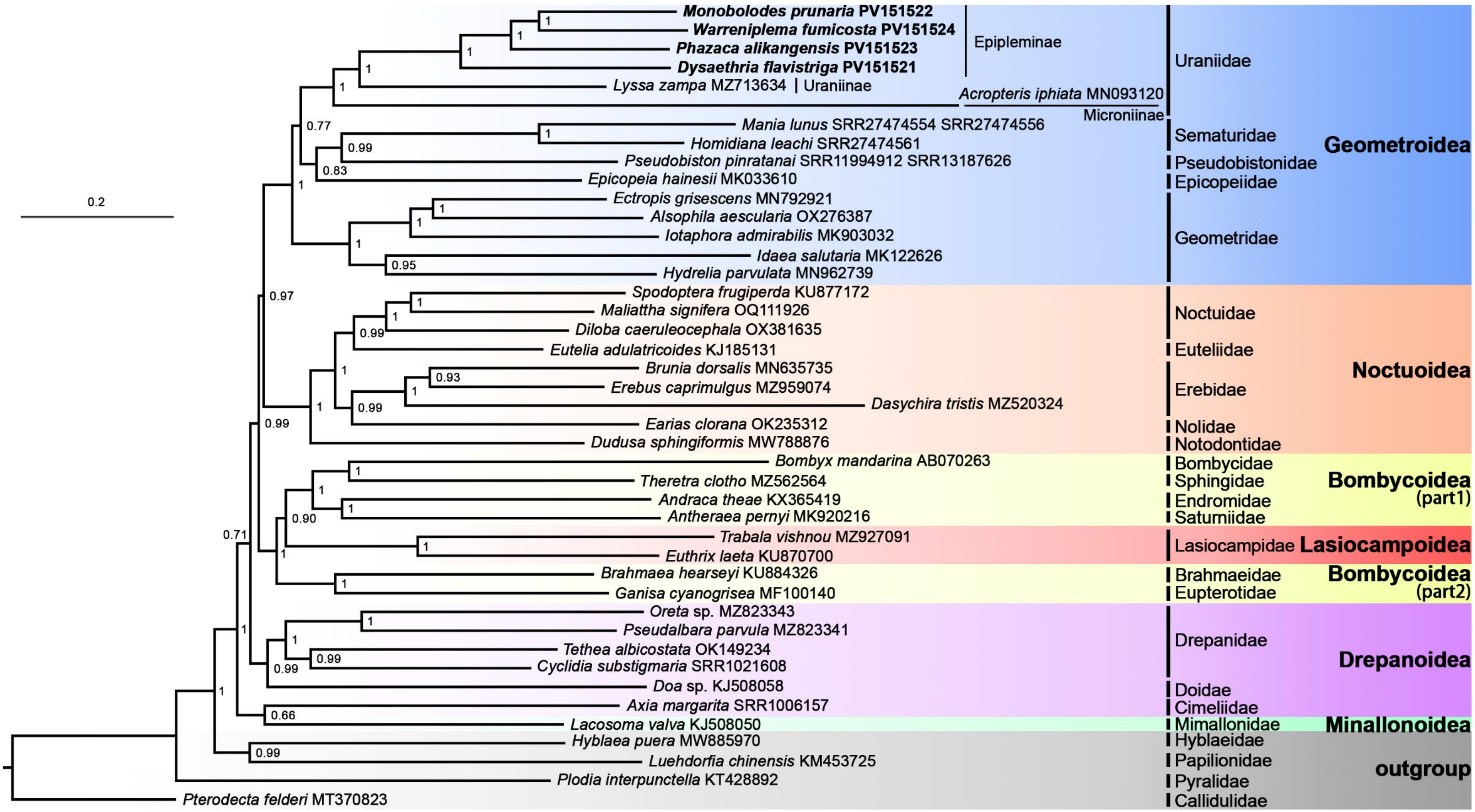

Figure S15. Phylogenetic tree of Macroheterocera produced by Bayesian Inference (BI) based on the PCG123 dataset. Numerals at nodes are Bayesian posterior probabilities (PP).

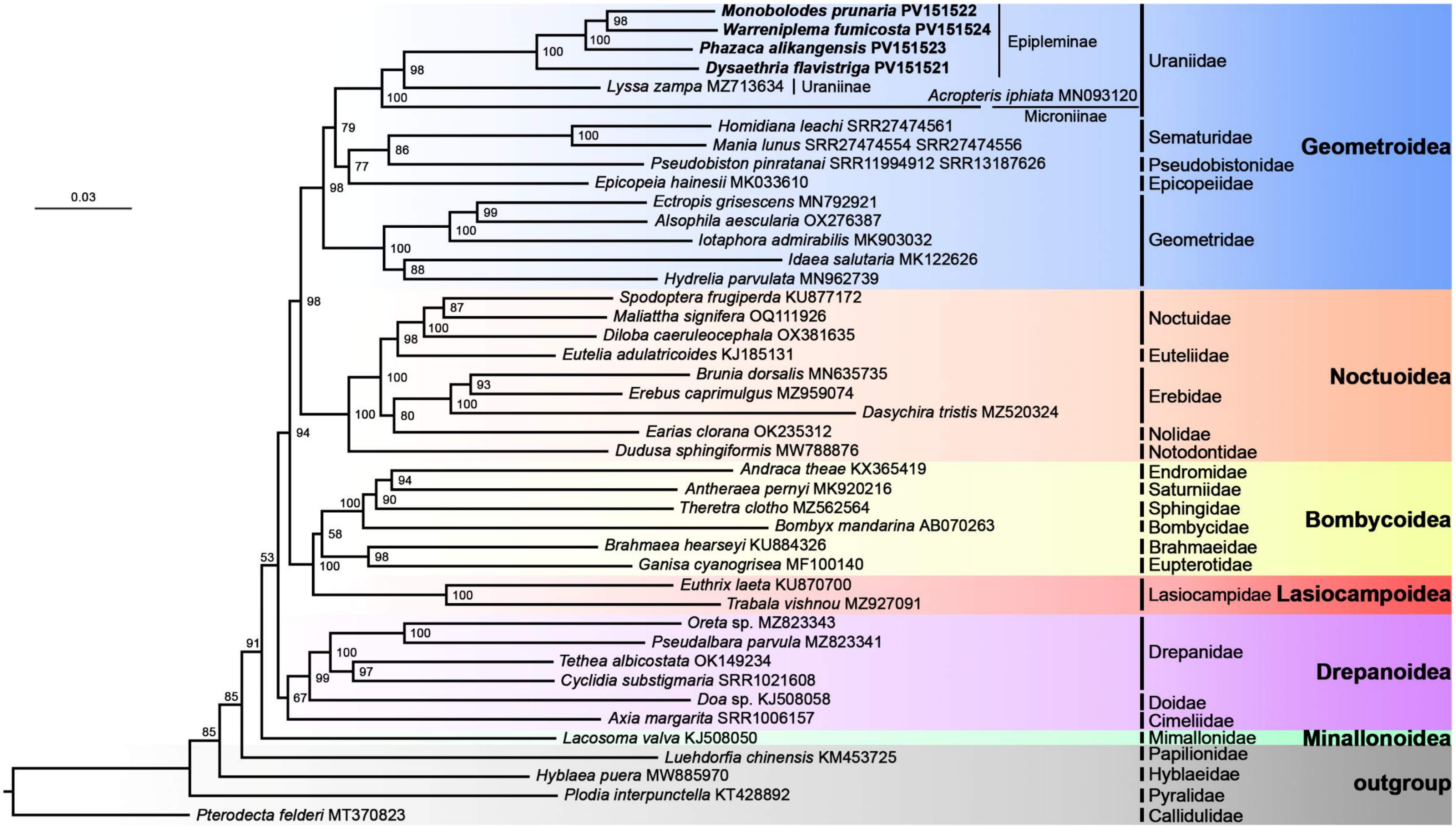

Figure S16. Phylogenetic tree of Macroheterocera produced by Maximum Likelihood (ML) based on the PCG12R dataset. Numerals at nodes are bootstrap support values (BS).

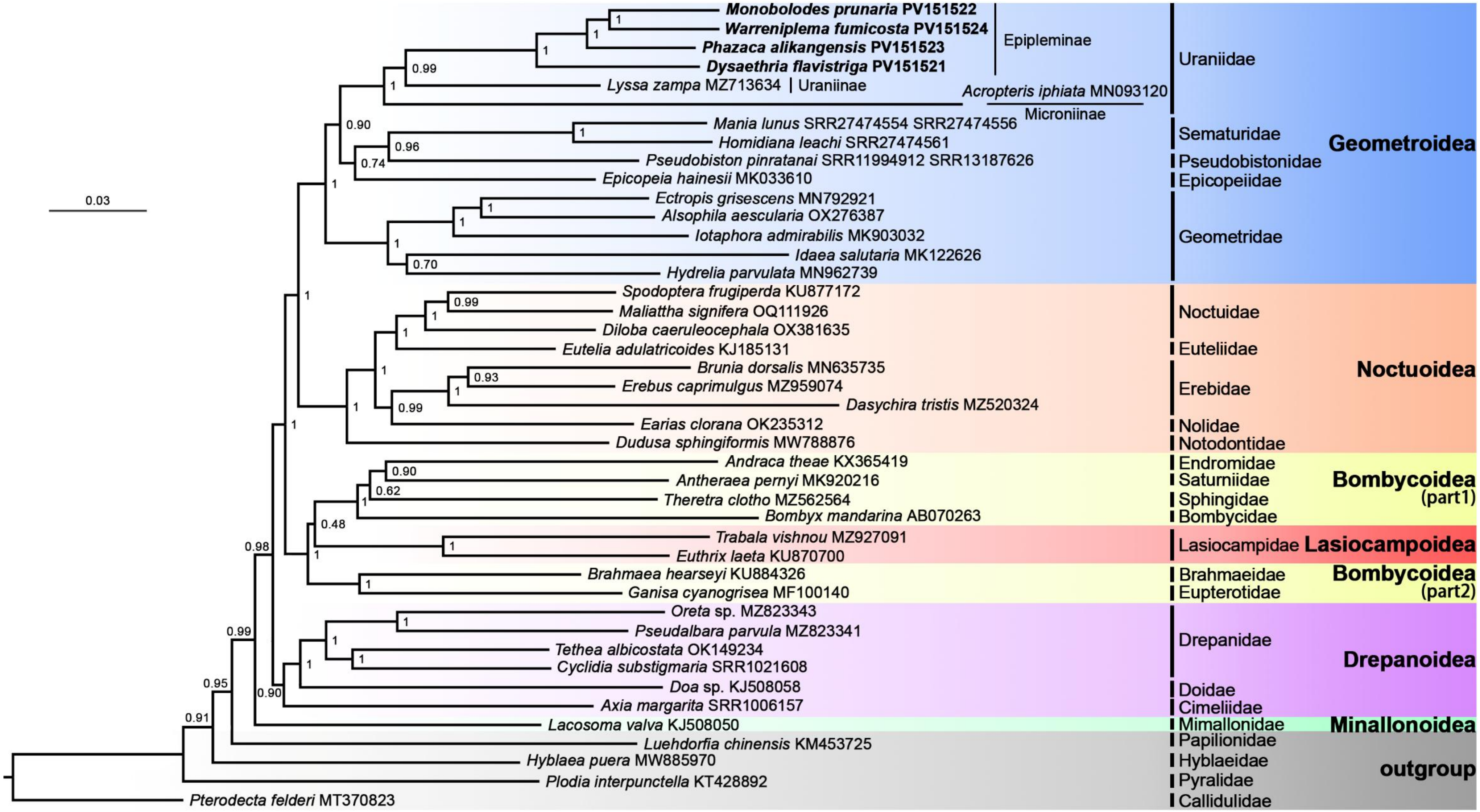

Figure S17. Phylogenetic tree of Macroheterocera produced by Bayesian Inference (BI) based on the PCG12R dataset. Numerals at nodes are Bayesian posterior probabilities (PP).

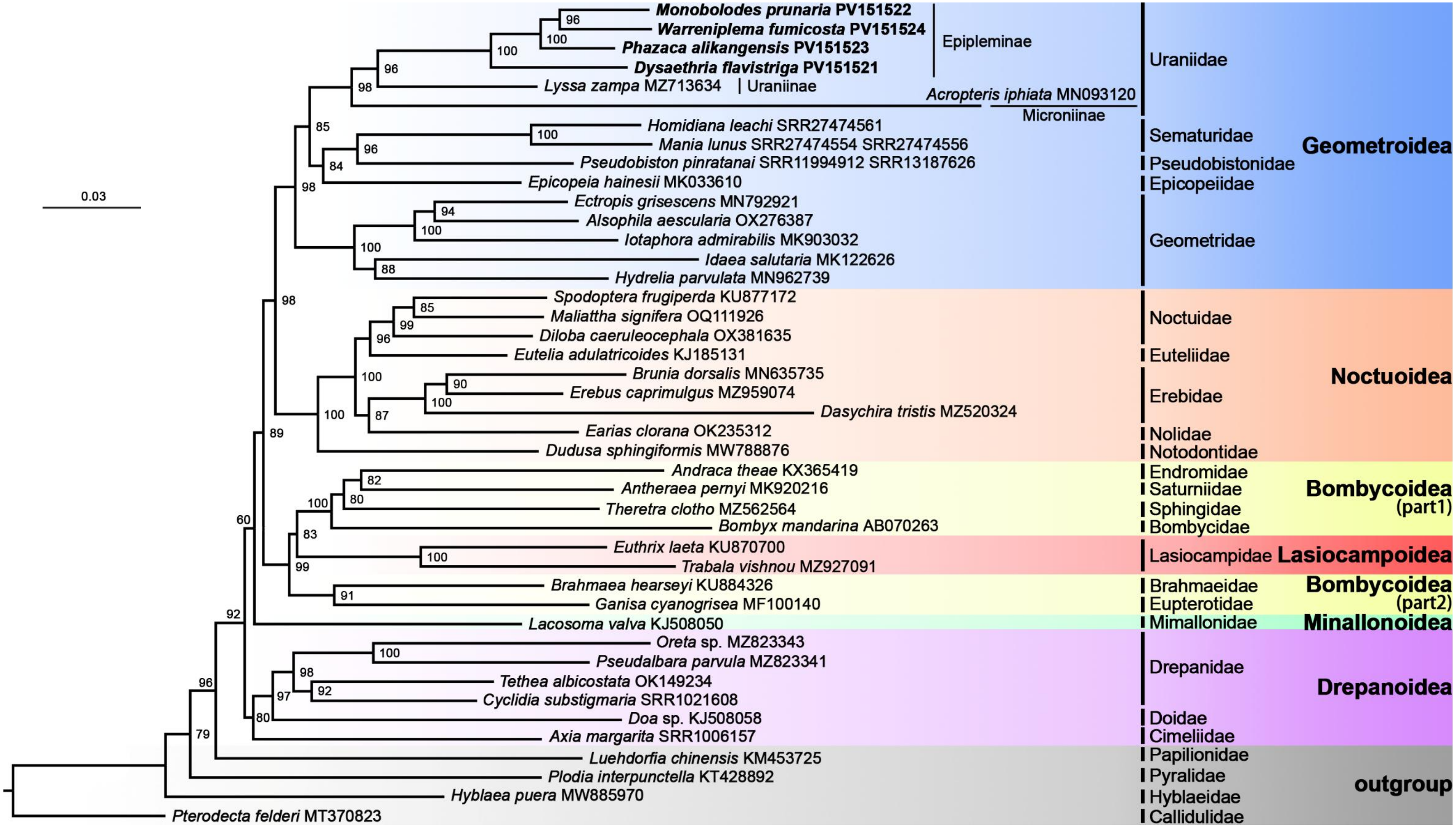

Figure S18. Phylogenetic tree of Macroheterocera produced by Maximum Likelihood (ML) based on the PCG12 dataset. Numerals at nodes are bootstrap support values (BS).

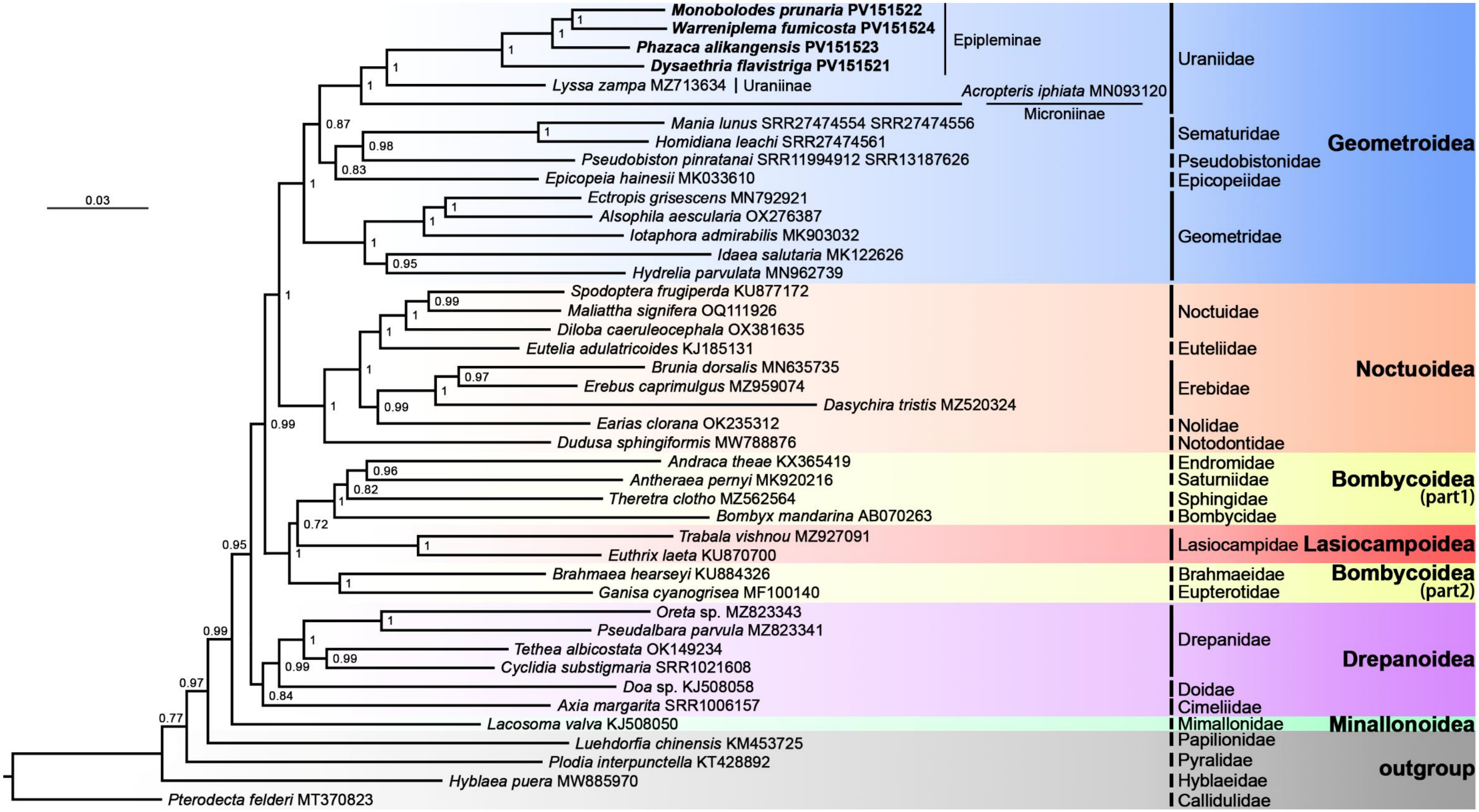

Figure S19. Phylogenetic tree of Macroheterocera produced by Bayesian Inference (BI) based on the PCG12 dataset. Numerals at nodes are Bayesian posterior probabilities (PP).

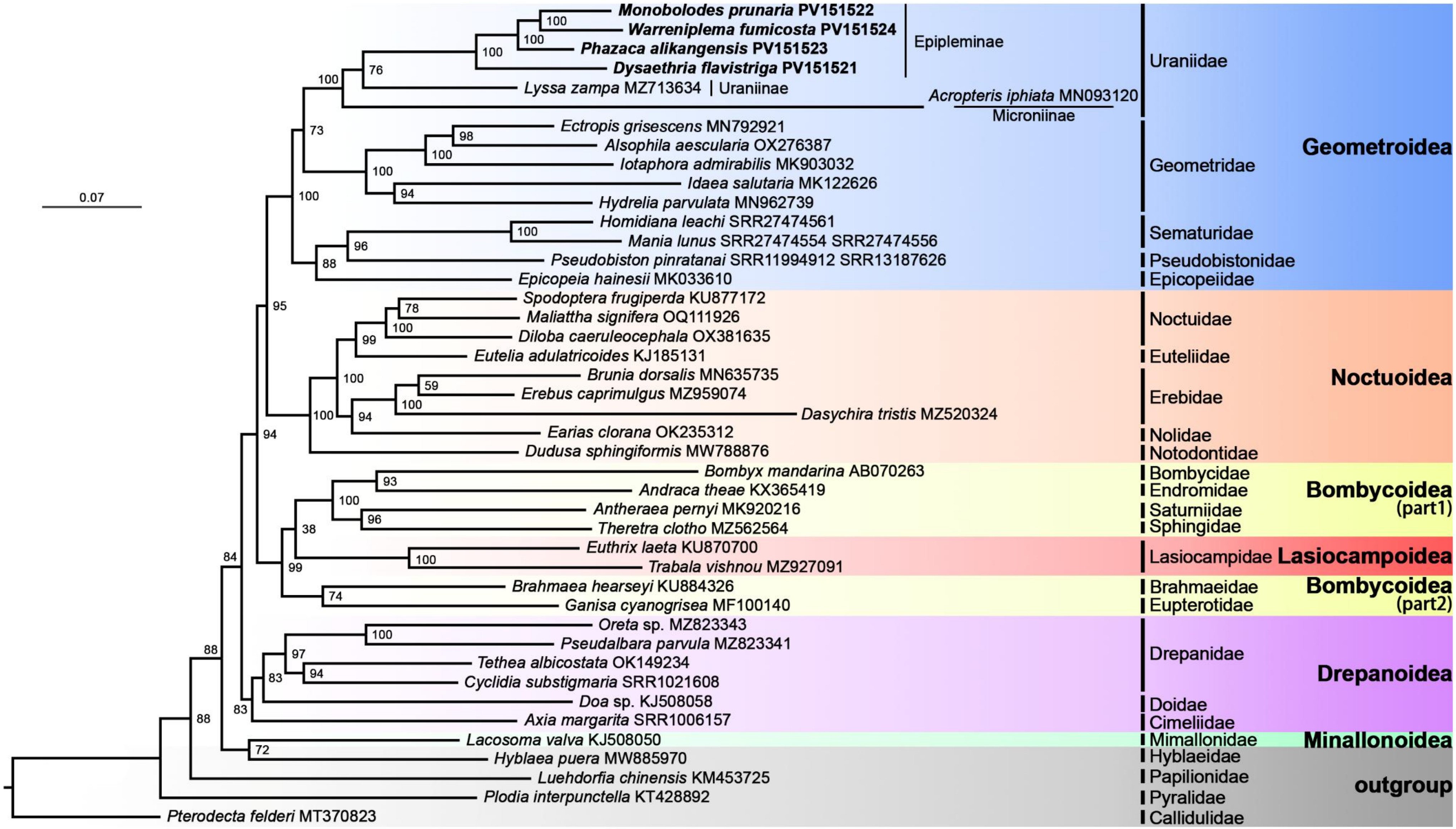

Figure S20. Phylogenetic tree of Macroheterocera produced by Maximum Likelihood (ML) based on the AA dataset. Numerals at nodes are bootstrap support values (BS).
