# Supplementary material for: ﻿The first mitogenomes of the subfamily Epipleminae (Lepidoptera, Uraniidae) and phylogenetic analysis of Macroheterocera
Source: Zookeys. 2025 Oct 15;1255:343–63. doi: 10.3897/zookeys.1255.164711 (PMC12547423; doi:10.3897/zookeys.1255.164711)
Supplement: Supplementary material 2 — Supplementary tables [file zookeys-1255-343_article-164711__-s002.pdf]

Table S1. Partitioning scheme and corresponding substitution models determined by ModelFinder for the PCG123R dataset in the ML analysis. The p1, p2 and p3 denote the first, second and third codon positions of each PCG, respectively.

**Partitions**	**Models**	**Data partitions**
P1	GTR+F+I+G4	atp6p1, cytbp1
P2	GTR+F+I+G4	atp6p2, cox1p2, cox2p2,
P3	K3Pu+F+I+G4	atp6p3, atp8p3, cox1p3, cox2p3, cox3p3, nd2p3, nd3p3, nd6p3
P4	TN+F+I+G4	atp8p1
P5	TPM3u+F+I+G4	atp8p2, nd6p2
P6	GTR+F+I+G4	cox1p1, cox2p1, cox3p1
P7	TVM+F+I+G4	cox3p2, cytbp2, nd2p2, nd3p2
P8	TIM2+F+I+G4	cytbp3
P9	TVM+F+I+G4	nd1p1, nd4p1, nd4lp1, nd5p1
P10	GTR+F+I+G4	nd1p2, nd4p2, nd4lp2, nd5p2
P11	TIM+F+I+G4	nd1p3, nd4p3
P12	GTR+F+I+G4	nd2p1, nd3p1
P13	TPM2u+F+G4	nd4lp3
P14	TIM2+F+I+G4	nd5p3
P15	TIM2+F+I+G4	nd6p1
P16	GTR+F+I+G4	*rrnS*
P17	GTR+F+I+G4	*rrnL*

Table S2. The partitioning scheme and corresponding substitution models determined by ModelFinder for the PCG123R dataset in the BI analysis. The p1, p2 and p3 denote the first, second and third codon positions of each PCG, respectively.

**Partitions**	**Models**	**Data partitions**
P1	GTR+F+I+G4	nd2p1, nd3p1
P2	GTR+F+I+G4	nd2p2, cox3p2, cytbp2, nd3p2, atp6p2
P3	GTR+F+G4	nd2p3
P4	GTR+F+I+G4	cox3p1, cox2p1, cox1p1
P5	GTR+F+G4	cox3p3, atp6p3
P6	GTR+F+I+G4	atp8p1
P7	GTR+F+I+G4	atp8p2, nd6p2
P8	GTR+F+I+G4	atp8p3, cox1p3, nd3p3, nd6p3
P9	GTR+F+I+G4	cox2p2, cox1p2
P10	GTR+F+G4	cox2p3
P11	GTR+F+I+G4	*rrnS*
P12	GTR+F+I+G4	cytbp1, atp6p1
P13	GTR+F+I+G4	cytbp3
P14	GTR+F+I+G4	nd4lp1, nd1p1, nd4p1, nd5p1
P15	GTR+F+I+G4	nd4lp2, nd1p2, nd4p2, nd5p2
P16	HKY+F+G4	nd4lp3
P17	GTR+F+G4	nd1p3, nd5p3
P18	GTR+F+I+G4	nd4p3
P19	GTR+F+I+G4	nd6p1
P20	GTR+F+I+G4	*rrnL*

Table S3. The partitioning scheme and corresponding substitution models determined by ModelFinder for the PCG123 dataset in the ML analysis. The p1, p2 and p3 denote the first, second and third codon positions of each PCG, respectively.

Partitions	Models	Data partitions
P1	GTR+F+I+G4	atp6p1, cytbp1, nd3p1
P2	GTR+F+I+G4	atp6p2, cox1p2, cox2p2
P3	GTR+F+G4	atp6p3, cox2p3, cox3p3
P4	TN+F+I+G4	atp8p1
P5	TPM3u+F+I+G4	atp8p2, nd6p2
P6	GTR+F+G4	atp8p3
P7	GTR+F+I+G4	cox1p1, cox2p1, cox3p1
P8	K3Pu+F+I+G4	cox1p3, nd3p3, nd6p3
P9	TVM+F+I+G4	cox3p2, cytbp2, nd2p2, nd3p2
P10	TIM2+F+I+G4	cytbp3
P11	TVM+F+I+G4	nd1p1, nd4p1, nd4lp1, nd5p1
P12	GTR+F+I+G4	nd1p2, nd4p2, nd4lp2, nd5p2
P13	K3Pu+F+I+G4	nd1p3, nd4p3
P14	TIM2+F+I+G4	nd2p1
P15	TIM+F+I+G4	nd2p3
P16	TPM2u+F+G4	nd4lp3
P17	TIM2+F+I+G4	nd5p3
P18	TIM2+F+G4	nd6p1

Table S4. The partitioning scheme and corresponding substitution models determined by ModelFinder for the PCG123 dataset in the BI analysis. The p1, p2 and p3 denote the first, second and third codon positions of each PCG, respectively.

Partitions	Models	Data partitions
P1	GTR+F+I+G4	cox1p1, cox3p1, cox2p1
P2	GTR+F+I+G4	cox1p2, cox2p2
P3	GTR+F+I+G4	cox1p3, nd6p3, nd3p3, atp8p3
P4	GTR+F+I+G4	nd4p1, nd5p1, nd4lp1, nd1p1
P5	GTR+F+I+G4	nd4p2, nd5p2, nd4lp2, nd1p2
P6	HKY+F+G4	nd4p3, nd4lp3
P7	GTR+F+I+G4	cox3p2, nd2p2, nd3p2, atp6p2, cytbp2
P8	GTR+F+G4	cox3p3, cox2p3
P9	GTR+F+G4	nd5p3, nd1p3
P10	GTR+F+I+G4	nd2p1
P11	HKY+F+I+G4	nd2p3, atp6p3, cytbp3
P12	GTR+F+G4	nd6p1
P13	GTR+F+I+G4	nd6p2, atp8p2
P14	GTR+F+I+G4	nd3p1, atp6p1, cytbp1
P15	GTR+F+I+G4	atp8p1

Table S5. The partitioning scheme and corresponding substitution models determined by ModelFinder for the PCG12R dataset in the ML analysis. The p1 and p2 denote the first and second codon positions of each PCG, respectively.

**Partitions**	**Models**	**Data partitions**
P1	GTR+F+I+G4	atp6p1, cox2p1, cytbp1
P2	TVM+F+I+G4	atp6p2, cox3p2, cytbp2, nd2p2, nd3p2
P3	TIM2+F+I+G4	atp8p1, nd6p1
P4	TIM2+F+I+G4	atp8p2, nd3p1
P5	GTR+F+I+G4	cox1p1
P6	TVM+F+I+G4	cox1p2, cox2p2
P7	GTR+F+I+G4	cox3p1
P8	GTR+F+I+G4	nd1p1, nd4p1, nd4lp1, nd5p1, *rrnS*
P9	GTR+F+I+G4	nd1p2, nd4p2, nd4lp2, nd5p2
P10	TIM2+F+I+G4	nd2p1
P11	TPM3u+F+I+G4	nd6p2
P12	GTR+F+I+G4	*rrnL*

Table S6. The partitioning scheme and corresponding substitution models determined by ModelFinder for the PCG12R dataset in the BI analysis. The p1 and p2 denote the first and second codon positions of each PCG, respectively.

**Partitions**	**Models**	**Data partitions**
P1	GTR+F+I+G4	cox1p1
P2	GTR+F+I+G4	cox1p2, cox2p2
P3	GTR+F+I+G4	nd4p1, nd4lp1, nd5p1, nd1p1, *rrnS*
P4	GTR+F+I+G4	nd4p2, nd4lp2, nd5p2, nd1p2
P5	GTR+F+I+G4	nd2p1
P6	GTR+F+I+G4	nd2p2, cytbp2, atp6p2, cox3p2, nd3p2
P7	GTR+F+I+G4	atp8p1, nd6p1
P8	GTR+F+I+G4	atp8p2, nd3p1
P9	GTR+F+I+G4	cytbp1, atp6p1
P10	GTR+F+I+G4	*rrnL*
P11	GTR+F+I+G4	cox2p1
P12	GTR+F+I+G4	cox3p1
P13	GTR+F+G4	nd6p2

Table S7. The partitioning scheme and corresponding substitution models determined by ModelFinder for the PCG12 dataset in the ML analysis. The p1 and p2 denote the first and second codon positions of each PCG, respectively.

**Partitions**	**Models**	**Data partitions**
P1	GTR+F+I+G4	atp6p1, cytbp1
P2	TVM+F+I+G4	atp6p2, cox2p2, cox3p2, cytbp2
P3	TIM2+F+I+G4	atp8p1, nd6p1
P4	TIM2+F+I+G4	atp8p2, nd2p1
P5	TIM2+F+I+G4	cox1p1
P6	TVM+F+I+G4	cox1p2
P7	TIM2+F+I+G4	cox2p1
P8	GTR+F+I+G4	cox3p1
P9	TVM+F+I+G4	nd1p1, nd4lp1, nd5p1
P10	GTR+F+I+G4	nd1p2, nd4p2, nd4lp2, nd5p2
P11	TVM+F+I+G4	nd2p2, nd3p2
P12	GTR+F+I+G4	nd3p1
P13	K3Pu+F+I+G4	nd4p1
P14	TPM3u+F+I+G4	nd6p2

Table S8. The partitioning scheme and corresponding substitution models determined by ModelFinder for the PCG12 dataset in the BI analysis. The p1 and p2 denote the first and second codon positions of each PCG, respectively.

**Partitions**	**Models**	**Data partitions**
P1	GTR+F+I+G4	atp8p1, nd6p1
P2	GTR+F+I+G4	atp8p2, nd2p1
P3	GTR+F+I+G4	cox1p1
P4	GTR+F+I+G4	cox1p2
P5	GTR+F+I+G4	nd2p2, nd3p2, cox3p2, cytbp2, atp6p2
P6	GTR+F+I+G4	nd3p1
P7	GTR+F+I+G4	nd1p1, nd5p1, nd4lp1
P8	GTR+F+I+G4	nd1p2, nd4p2, nd5p2, nd4lp2
P9	GTR+F+G4	nd6p2
P10	GTR+F+I+G4	cox3p1
P11	GTR+F+I+G4	cytbp1, atp6p1
P12	GTR+F+I+G4	nd4p1
P13	GTR+F+I+G4	cox2p1
P14	GTR+F+I+G4	cox2p2

Table S9. The partitioning scheme and corresponding substitution models determined by ModelFinder for the AA dataset in the ML analysis.

**Partitions**	**Models**	**Data partitions**
P1	mtART+F+I+G4	atp6, nd2, nd3
P2	Q.plant+F+G4	atp8
P3	mtART+I+G4	cox1
P4	mtInv+I+G4	cox2, cox3, cytb
P5	mtART+F+I+G4	nd1, nd4l
P6	mtZOA+F+I+G4	nd4, nd5
P7	mtART+F+G4	nd6

Table S10. Annotation of the mitochondrial genomes of four Epiplemine species: *D. flavistriga*/*M. prunaria*/*P. alikangensis*/*W. fumicosta*.

Gene	Position		Size (bp)	Codon		IGN (bp)	Strand
	From	To		Start	Stop		
trnM	1/1/<br>1/1	68/67/<br>68/68	68/67/<br>68/68	—	—	0/0/<br>0/0	J/J/<br>J/J
trnI	69/70/<br>69/69	134/134/<br>133/133	66/65/<br>65/65	—	—	0/2/<br>0/0	J/J/<br>J/J
trnQ	132/132/<br>131/131	200/200/<br>199/199	69/69/<br>69/69	—	—	-3/-3/<br>-3/-3	N/N/<br>N/N
ND2	257/255/<br>300/257	1270/1268/<br>1313/1270	1014/1014/<br>1014/1014	ATT/ATT/<br>ATC/ATT	TAA/TAA/<br>TAA/TAA	56/54/<br>100/57	J/J/<br>J/J
trnW	1269/1268/<br>1313/1269	1335/1333/<br>1378/1334	67/66/<br>66/66	—	—	-2/-1/<br>-1/-2	J/J/<br>J/J
trnC	1328/1326/<br>1371/1327	1395/1393/<br>1438/1395	68/68/<br>68/69	—	—	-8/-8/<br>-8/-8	N/N/<br>N/N
trnY	1400/1402/<br>1445/1400	1465/1466/<br>1510/1464	66/65/<br>66/65	—	—	4/8/<br>6/4	N/N/<br>N/N
COX1	1469/1478/<br>1517/1482	2999/3008/<br>3047/3012	1531/1531/<br>1531/1531	CGA/CGA/<br>CGA/CGA	T/T/<br>T/T	3/11/<br>6/17	J/J/<br>J/J
trnL2	3000/3009/<br>3048/3013	3066/3075/<br>3114/3079	67/67/<br>67/67	—	—	0/0/<br>0/0	J/J/<br>J/J
COX2	3067/3076/<br>3115/3080	3748/3757/<br>3796/3761	682/682/<br>682/682	ATG/ATG/<br>ATG/ATG	T/T/<br>T/T	0/0/<br>0/0	J/J/<br>J/J
trnK	3749/3758/<br>3797/3762	3819/3828/<br>3867/3832	71/71/<br>71/71	—	—	0/0/<br>0/0	J/J/<br>J/J
trnD	3820/3830/<br>3870/3832	3886/3895/<br>3935/3899	67/66/<br>66/68	—	—	0/1/<br>2/-1	J/J/<br>J/J
ATP8	3887/3896/<br>3936/3900	4045/4054/<br>4094/4058	159/159/<br>159/159	ATT/ATC/<br>ATA/ATC	TAA/TAA/<br>TAA/TAA	0/0/<br>0/0	J/J/<br>J/J
ATP6	4039/4048/<br>4088/4052	4716/4725/<br>4765/4729	678/678/<br>678/678	GTG/ATG/<br>ATG/ATG	TAA/TAA/<br>TAA/TAA	-7/-7/<br>-7/-7	J/J/<br>J/J
COX3	4716/4730/<br>4765/4734	5504/5518/<br>5553/5522	789/789/<br>789/789	ATG/ATG/<br>ATG/ATG	TAA/TAA/<br>TAA/TAA	-1/4/<br>-1/4	J/J/<br>J/J
trnG	5507/5521/<br>5556/5525	5573/5586/<br>5622/5591	67/66/<br>67/67	—	—	2/2/<br>2/2	J/J/<br>J/J
ND3	5574/5587/<br>5623/5592	5927/5940/<br>5976/5945	354/354/<br>354/354	ATT/ATT/<br>ATT/ATT	TAA/TAA/<br>TAA/TAA	0/0/<br>0/0	J/J/<br>J/J
trnA	5941/5972/<br>6003/5972	6005/6037/<br>6067/6036	65/66/<br>65/65	—	—	13/31/<br>26/26	J/J/<br>J/J
trnR	6005/6038/<br>6067/6040	6068/6100/<br>6129/6102	64/63/<br>63/63	—	—	-1/0/<br>-1/3	J/J/<br>J/J
trnN	6082/6112/<br>6138/6103	6147/6177/<br>6202/6168	66/66/<br>65/66	—	—	13/11/<br>8/0	J/J/<br>J/J
trnS1	6149/6177/<br>6201/6167	6214/6239/<br>6262/6228	66/63/<br>62/62	—	—	1/-1/<br>-2/-2	J/J/<br>J/J
trnE	6218/6264/<br>6295/6239	6283/6330/<br>6361/6304	66/67/<br>67/66	—	—	3/24/<br>32/10	J/J/<br>J/J
trnF	6284/6333/	6350/6398/	67/66/	—	—	0/2/	N/N/

	6365/6310	6431/6375	67/66			3/5	N/N
ND5	6351/6411/	8097/8153/	1747/1743/	ATT/ATT/	T/TAA/	0/12/	N/N/
	6438/6384	8183/8126	1746/1743	ATA/ATT	TAA/TAA	6/8	N/N
trnH	8098/8154/	8163/8218/	66/65/	—	—	0/0/	N/N/
	8184/8127	8248/8191	65/65			0/0	N/N
ND4	8164/8219/	9502/9557/	1339/1339/	ATG/ATG/	T/T/	0/0/	N/N/
	8248/8192	9588/9530	1341/1339	ATG/ATG	TAA/T	-1/0	N/N
ND4L	9508/9564/	9792/9857/	285/294/	ATG/ATG/	TAA/TAA/	5/6/	N/N/
	9589/9530	9876/9823	288/294	ATG/ATG	TAA/TAA	0/-1	N/N
trnT	9801/9864/	9865/9930/	65/67/	—	—	8/6/	J/J/
	9883/9828	9949/9893	67/66			6/4	J/J
trnP	9866/9931/	9929/9994/	64/64/	—	—	0/0/	N/N/
	9950/9894	10013/9957	64/64			0/0	N/N
ND6	9938/9997/	10468/10524/	531/528/	ATA/ATA/	TAA/TAA/	8/2/	J/J/
	10016/9960	10546/10484	531/525	ATA/ATA	TAA/TAA	2/2	J/J
CYTB	10481/10542/	11632/11693/	1152/1152/	ATG/ATG/	TAA/TAA/	12/17/	J/J/
	10599/10509	11750/11660	1152/1152	ATG/ATG	TAA/TAA	52/24	J/J
trnS2	11635/11697/	11698/11762/	64/66/	—	—	2/3/	J/J/
	11759/11659	11825/11723	67/65			8/-2	J/J
ND1	11719/11781/	12657/12717/	939/937/	ATG/TTG/	TAA/T/	20/18/	N/N/
	11844/11746	12780/12684	937/939	TTG/TTG	T/TAG	18/22	N/N
trnL1	12659/12718/	12731/12787/	73/70/	—	—	1/0/	N/N/
	12781/12685	12849/12755	69/71			0/0	N/N
rrnL	12732/12788/	14125/14187/	1394/1400/	—	—	0/0/	N/N/
	12850/12756	14261/14213	1412/1458			0/0	N/N
trnV	14126/14188/	14190/14253/	65/66/	—	—	0/0/	N/N/
	14262/14214	14328/14277	67/64			0/0	N/N
rrnS	14191/14254/	14973/15032/	783/779/	—	—	0/0/	N/N/
	14329/14278	15111/15056	783/779			0/0	N/N
CR	14974/15033/	15404/15258/	431/226/	—	—	0/0/	J/J/
	15112/15057	15482/15467	371/411			0/0	J/J
